# Rapid Cooling After Device Annealing Suppresses Interface Losses in Cu_2_ZnSnS_4_ Solar Cells Enabling High Efficiency and Stability

**DOI:** 10.1002/advs.77574

**Published:** 2026-09-06

**Authors:** Achmad Nasyori, Mati Danilson, Idil Mengü, Maris Pilvet, Jüri Krustok, Reelika Kaupmees, Valdek Mikli, Raavo Josepson, Yuancai Gong, Akhil Alexander, Shreyash S. Hadke, Aurelian Catalin Galca, Paola Vivo, Lydia Helena Wong, Thomas Unold, Maarja Grossberg‐Kuusk, Marit Kauk‐Kuusik

**Affiliations:** ^1^ Department of Materials and Environmental Technology Tallinn University of Technology Tallinn Estonia; ^2^ Division of Physics Tallinn University of Technology Tallinn Estonia; ^3^ Photovoltaic Lab Micro and Nano Technologies Group Electronic Engineering Department Barcelona East School of Engineering Universitat Politècnica de Catalunya Barcelona Spain; ^4^ Barcelona Research Center for Multiscale Science and Engineering Universitat Politècnica de Catalunya Barcelona Spain; ^5^ Hybrid Solar Cells Faculty of Engineering and Natural Sciences Tampere University Tampere Finland; ^6^ Department of Materials Science and Engineering Northwestern University Evanston Illinois USA; ^7^ Laboratory of Complex Heterostructures and Multifunctional Materials (HeCoMat) National Institute of Materials Physics Magurele Ilfov Romania; ^8^ International Centre for Advanced Training and Research in Physics Magurele Ilfov Romania; ^9^ School of Materials Science and Engineering Nanyang Technological University Singapore Singapore; ^10^ Department Structure and Dynamics of Energy Materials Helmholtz‐Zentrum Berlin für Materialien Und Energie Lise‐Meitner Campus Berlin Germany

**Keywords:** back‐contact losses, cooling engineering, Cu_2_ZnSnS_4_, device annealing, heterojunction, outdoor and indoor photovoltaic applications

## Abstract

Wide‐bandgap kesterite (Cu_2_ZnSnS_4_, CZTS) is an earth‐abundant and environmentally friendly absorber, making it a promising candidate for both outdoor and indoor photovoltaic applications. However, device performance remains limited by multiple loss mechanisms, including recombination at the CdS/CZTS heterojunction and resistive back‐contact losses at the Mo/CZTS interface. Here, we report solution‐processed CZTS devices in which controlling the cooling dynamics after device annealing in ambient air provides an effective and scalable strategy to overcome these limitations and enable stable, high‐performance devices. By implementing a rapid cooling process (RCP), we suppress non‐radiative recombination at the CdS/CZTS heterojunction, resulting in a decrease in saturation current density (*J_0_*) by three orders of magnitude. Moreover, RCP improves the Mo/CZTS back contact by modifying the MoS_2_‐related region, which may facilitate hole extraction and reduce back‐contact losses. As a result of these combined interface improvements, RCP‐treated devices retain a PCE of 11.1% after ∼1500 h of damp heat aging at 85°C, with a *Voc* corresponding to 62.4% of the thermodynamic limit. The RCP device also delivers an indoor PCE of 15.9% under 2700 K LED illumination, representing the highest reported performance for kesterite solar cells under indoor conditions at 1000 lux.

## Graphical abstract

Rapid cooling after device annealing regulates the CdS/CZTS heterojunction and Mo/CZTS back contact, suppressing interfacial recombination and modifying the interfacial MoS_2_ layer, thereby enabling a 15.9% indoor PCE in pure‐sulfide CZTS solar cells.

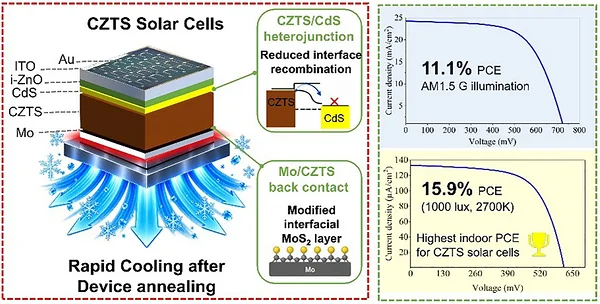

## 1 Introduction

Indoor photovoltaics (IPVs) are gaining increasing attention as a promising power source for self‐powered IoT devices. By converting ambient indoor light into electricity, they offer a sustainable alternative to conventional batteries [1]. Among emerging IPV technologies, kesterite (Cu_2_ZnSnS_4_, CZTS) has attracted considerable interest as an earth‐abundant and environmentally benign photovoltaic material due to its low cost, non‐toxicity, stable crystal structure, and compatibility with large‐scale manufacturing. CZTS also offers a tunable direct bandgap of 1.5–2.0 eV when alloyed with Ge or Ag, together with a high absorption coefficient. These properties make CZTS suitable for single‐junction and tandem photovoltaic devices, with strong potential for indoor photovoltaics and IoT applications [2, 3, 4, 5]. Despite these advantages, the highest certified efficiencies of pure‐sulfide CZTS solar cells under standard outdoor conditions (AM1.5G) are 11.9% for solution‐processed devices and 12.4% for sputtered devices remain well below the theoretical thermodynamic (Shockley–Queisser) limit, indicating persistent challenges associated with absorber bulk quality, deep level defects, interface recombination at the CdS/CZTS heterojunction, and back contact losses [6, 7, 8, 9]. To address these issues, various optimization strategies have been investigated including molecular ink design, sulfurization/selenization strategies, bandgap engineering [10], the development of alternative buffer layers [11], and heterojunction annealing or full device annealing [7, 8, 12, 13].

Among the various optimization strategies, heterojunction annealing and device annealing have emerged as some of the most effective and scalable approaches for enhancing the performance of kesterite solar cells [12, 14, 15, 16, 17]. In general, annealing is applied after deposition of the CdS buffer layer [14, 15], whereas device annealing is performed after completion of the full device stack [12, 16, 17]. For example, Hao et al. [15] reported that annealing of CZTS/CdS (270°C for 10 min, N_2_) improved sputtered CZTS efficiency from 7.8% to 11.0% via reduced recombination and enhanced interdiffusion. Xin et al. [18] further showed that prolonged low‐temperature annealing promotes Cd/Zn redistribution and junction grading, enabling 13.0% PCE in Ag‐alloyed CZTSSe devices. Other studies reported that device annealing at 300°C can yield Cd‐alloyed CZTS with efficiencies of up to 12.6% by enhancing interdiffusion and improving ITO window‐layer properties [16]. However, heat treatment above a critical temperature (> 260°C) increases cation disorder (i.e., mixing of Cu and Zn atoms in the crystal lattice), which reduces the bandgap and enhance *J_SC_* [17, 19, 20]. More recently, annealing in hydrogen‐ and oxygen‐rich environments has been shown to further suppress deep‐level defects associated with sulfur vacancies and enhance carrier collection, resulting in certified CZTS efficiencies exceeding 11.4% and 11.51% [7, 8]. These studies have established heterojunction modification and device annealing as powerful and versatile approaches for suppressing recombination losses, minimizing deep‐level defects, optimizing heterojunction quality, modifying order‐disorder state, improving window‐layer properties, and thereby enhancing carrier collection and overall device performance in kesterite solar cells [7, 8, 14, 15, 16, 17, 18].

However, most prior studies have focused on optimizing annealing temperature, duration, and ambient atmosphere, while the influence of cooling dynamics after device annealing has received comparatively limited attention in CZTS solar cells. In cadmium telluride (CdTe) photovoltaics, rapid cooling (quenching) is a critical step for stabilizing high‐temperature defect configurations by freezing electrically active dopants into substitutional lattice sites, thereby maximizing carrier concentration at room temperature [21, 22]. It has been found that cooling kinetics strongly influence elemental diffusion, defect redistribution, and interfacial stability; in particular, rapid cooling suppresses defect segregation, sustains high hole densities, and shifts the Fermi level closer to the valence band maximum (VBM) [21, 22]. Nevertheless, intensive studies of the effects of cooling dynamic after device annealing on CZTS device performance remain largely unexplored.

In this work, we introduce controlling the cooling dynamics after device annealing as an effective strategy to improve both efficiency and stability of wide‐bandgap CZTS solar cells for outdoor and indoor conditions. Devices processed with a rapid cooling process (RCP) achieved a champion PCE of 11.1% after stability measurement. Detailed electrical and optical characterizations showed that RCP suppresses interface recombination at the CZTS/CdS heterojunction, reduces the open‐circuit voltage deficit (*V_OC, def_*), and significantly decreases saturation current density *J_0_*. In addition to junction improvements, controlled cooling influences the Mo/CZTS back interface by modifying the thickness and crystallographic texture of the interfacial MoS_2_ layer, which may reduce back‐contact losses. We find that the RCP device reached an indoor PCE of 15.9% under 2700 K LED illumination at 1000 lux, representing the highest reported performance for CZTS solar cells under indoor conditions at 1000 lux. Overall, this study identifies cooling dynamics as a critical yet often overlooked processing parameter for advancing the efficiency and operational stability of CZTS photovoltaics in both outdoor and indoor conditions.

## 2 Results and Discussion

In our preceding study [12], we established a glovebox‐free fabrication route for solution‐processed CZTS solar cells using DMSO‐based precursors under ambient air, and demonstrated that post‐annealing of the complete Mo/CZTS/CdS/i‐ZnO/ITO device stack at 300°C for 12 min in ambient air using a closed graphite enclosure markedly improves fill factor and power conversion efficiency, yielding a champion PCE of 9.4%. These gains were attributed to reduced series resistance, improved CdS buffer layer crystallinity, and partial suppression of interface recombination at the CdS/CZTS heterojunction. Nevertheless, temperature‐dependent VOC analysis revealed that the activation energy (E_A_ = 1038 meV) remained below the absorber bandgap, confirming that interface recombination still constituted the dominant recombination pathway. Back‐contact losses and the role of the interfacial MoS_2_ layer were not addressed, device stability was not evaluated, and a single unoptimized cooling condition was applied. The present study addresses these open questions.

As a first step, we sought to improve the absorber quality relative to reference [12] by optimizing the sulfurization temperature profile. In our previous study [12], a single‐ramp profile (from Room Temperature to 650°C in ∼32.5 min, 20 min dwell, Ar atmosphere) was used; this protocol is retained here as Profile‐1 and serves as the direct internal reference condition. Three additional profiles were examined (Profiles 2–4), each introducing an intermediate dwell prior to the final high‐temperature step (details in Note S1 and Figures S1–S5). Among these, Profile‐2 — involving heating from room temperature to 575°C within 27 min with a 10 min dwell, followed by further heating to 650°C with a 20 min dwell — yielded the best results. Raman spectroscopy and XRD analysis revealed no significant differences in phase composition or crystal structure among the four profiles: all films display the characteristic kesterite CZTS A_1_ vibrational mode and the expected diffraction planes, with no detectable change in secondary phase content. The performance differences therefore originate from microstructural rather than phase‐compositional changes. SEM analysis showed that Profile‐2 produced the largest average lateral grain size (0.74 µm), a compact and pinhole‐free morphology with pronounced grain coalescence. AFM analysis further revealed that Profile‐2 exhibited lowest overall surface roughness among all profiles, with *R_q_* = 49.5 nm and *R_a_* = 33.7 nm. We attribute this to the intermediate 575°C dwell allowing more homogeneous nucleation and lateral grain coalescence before the 650°C step drives rapid grain boundary closure. The Profile‐2 film also exhibited a desirable Cu‐poor/Zn‐rich composition close to the targeted precursor ratio. Consistent with the improved microstructure, Profile‐2 devices achieved the highest *V_OC_* and PCE among all profiles, with a pre‐annealing baseline PCE of 7.3% (*J_SC_* = 20 mA·cm^−^^2^, *V_OC_* = 721 mV, FF = 50.5%) — substantially exceeding the 4.1% obtained with Profile‐1, the protocol of reference [12]. This improvement establishes Profile‐2 as the optimized absorber baseline for the subsequent cooling‐rate investigation.

### 2.1 The Effect of Device Annealing and Cooling Process

We then applied device annealing following the same protocol established in our previous work [12]: in ambient air on a hotplate, using a graphite enclosure at 300°C for 12 min (Figure S7). To confirm that 300°C remains the optimal annealing temperature with the new Profile‐2 absorber, we annealed devices at 100°C, 200°C, 250°C, 300°C, and 350°C for 12 min, each followed by a moderate cooling (∼4 min). Devices without annealing are denoted as the reference (“Ref”), while annealed devices are labeled according to their annealing temperature. Statistical photovoltaic parameters and extracted electronic characteristics are shown in Figure S8 and summarized in Table S2.

Device annealing at 100°C–200°C degraded all key PV parameters, indicating that these temperatures are insufficient to activate the diffusion‐driven bulk and interfacial modifications required for performance improvement [10, 14, 23]. Consistent with prior reports, effective device annealing is typically achieved above ∼200°C for short annealing durations of 5–15 min [6, 7, 10, 12, 13, 14, 15, 22, 23, 24, 25, 26, 27, 28, 29, 30]. In contrast, annealing at 300°C markedly increased PCE from 7.3% to 9.8%, driven by higher *J_SC_* (+1.7 mA·cm^−^^2^) and FF (from 50.5% to 62.9%), with correlated EQE improvements in both the 350–500 nm and 750–850 nm ranges (Figure S9). Raman spectra collected across the annealing series (Figure S10) show a distinct CdS peak appearing after annealing at 100°C and increasing steadily up to 350°C, indicating progressive crystallization of CdS from an initially amorphous phase, consistent with prior literatures [12, 27, 30, 31, 32]. Although higher CdS crystallinity generally benefits junction formation, excessive annealing temperatures adversely impacted FF. Annealing at 300°C provided the best trade‐off between CdS crystallinity and heterojunction quality, yielding reproducible PCE values above 9.0%, consistent with previous work [12, 15, 16, 27]. We therefore selected 300°C as the fixed annealing temperature for all subsequent cooling‐process studies.

**FIGURE 1:**
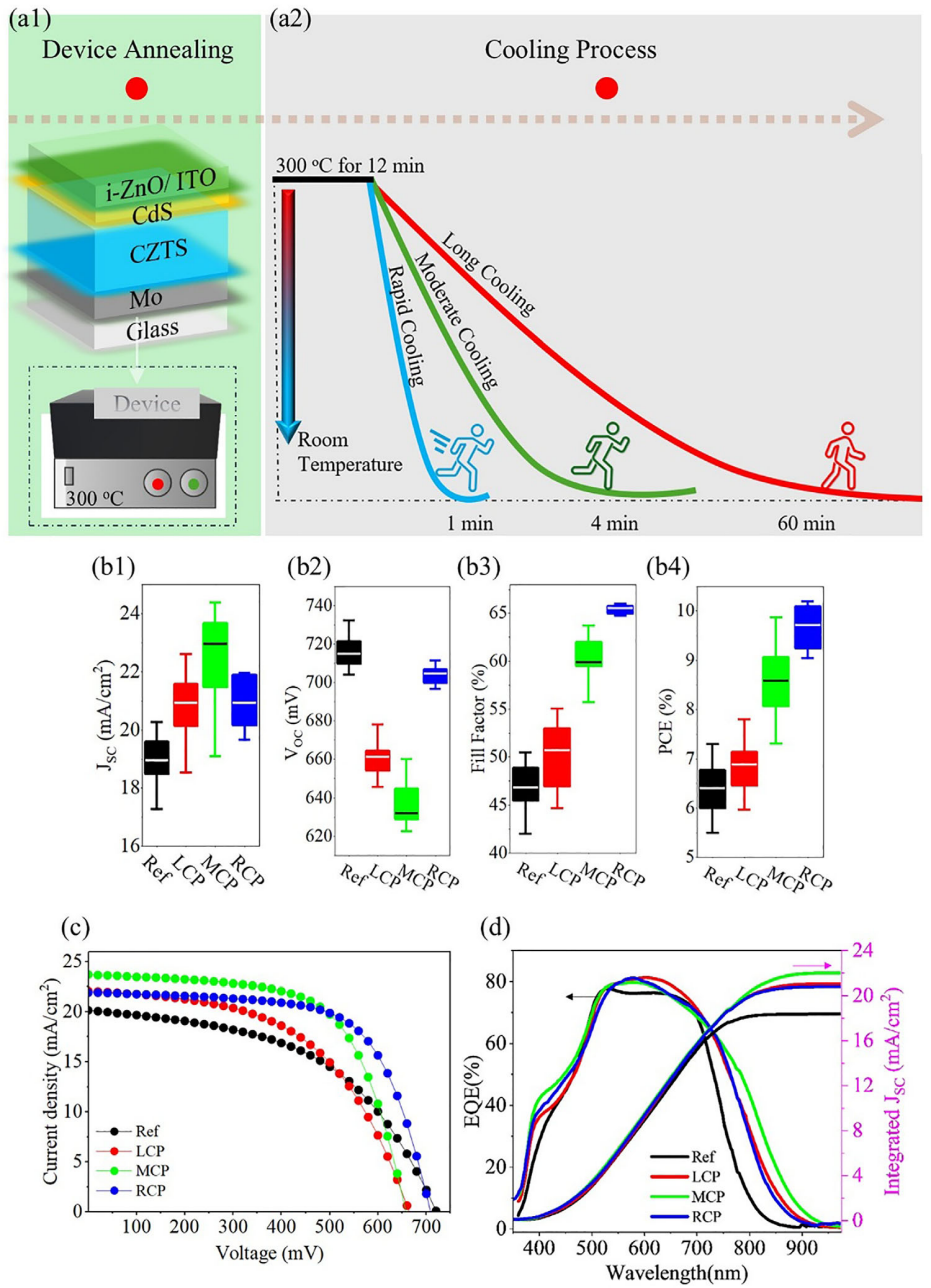
(a1–a3) Schematic illustration of the structure CZTS device, device annealing, and cooling processes: LCP, MCP, and RCP. (b1–b4) Statistical distributions of the photovoltaic parameters (*J_SC_*, *V_OC_*, *FF*, and *PCE*) for the Ref, LCP, MCP, and RCP devices. (c) *J–V* characteristics of the champion devices under each cooling condition. (d) EQE results and corresponding integrated *J_SC_* values.

To investigate whether cooling dynamics after annealing can push performance beyond what the annealing temperature alone determines, we systematically varied the cooling rate while keeping annealing conditions fixed at 300°C for 12 min. Three distinct cooling approaches were investigated: a long cooling process (LCP), a moderate cooling process (MCP), and a rapid cooling process (RCP). The LCP corresponds to natural cooling together with the hotplate, requiring approximately 60 min to reach room temperature. The MCP involves transferring the annealed sample and allowing cooling using a hand‐held paper sheet placed over a metal plate, resulting in a total cooling time of ∼4 min — equivalent to the single cooling condition applied throughout reference [12]. The RCP is achieved by immediately placing the annealed sample onto a metal plate at room temperature, enabling cooling within approximately ∼1 min (Figure 1a2). Cooling duration was measured with a surface thermocouple until the sample reached room temperature (∼25°C). Figure 1a1–a2 illustrates the device structure, the annealing procedure, and the three cooling steps schematically.

To investigate the electronic properties, we measured the photovoltaic characteristics of the Ref CZTS device (without annealing) and the devices subjected to the LCP, MCP, and RCP under standard AM 1.5G illumination. Statistical distributions of the PV parameters derived from 16 individual cells per device are shown in Figure 1b1–b4, ensuring reproducibility. All cooling approaches resulted in pronounced improvements in the distributions of *J_SC_*, *FF*, and PCE relative to the Ref device, confirming that the cooling pathway following device annealing strongly influences device performance. However, a modest reduction in *V_OC_* is observed for all annealed samples. Interestingly, despite this overall trend, the RCP devices exhibit a higher *V_OC_* than the LCP and MCP devices, resulting in the smallest *V_OC, def_* among the cooling conditions. This behavior could indicate improved junction quality by rapid cooling. Figure 1c shows current density−voltage (*J–V)* curves for the Ref, LCP, MCP, and RCP devices, with the corresponding PV parameters summarized in Table 1. The Ref device exhibits a PCE of 7.3%, with a *FF* of 50.5%, *J_SC_* of 20.0 mA·cm^−^^2^, and *V_OC_* of 721 mV. In comparison, the LCP device reaches a PCE of 7.8%, benefiting from increases in *FF* (53.4%) and *J_SC_* (22.0 mA·cm^−^^2^), accompanied by a reduced *V_OC_* of 664 mV. The MCP device exhibits further enhancement, achieving a PCE of 9.8%, with an *FF* of 62.9%, a *V_OC_* of 658 mV, and a *J_SC_* of 23.7 mA·cm^−^^2^. The RCP device yields the highest performance, attaining a champion PCE of 10.2%, with an *FF* of 66.1%, *J_SC_* of 21.8 mA·cm^−^^2^, and a *V_OC_* of 708 mV. These results highlight the effectiveness of rapid thermal quenching in preserving the beneficial effects of device annealing, while mitigating voltage losses, thereby enabling simultaneous improvements in current collection, *FF*, and overall device efficiency.

**TABLE 1:** Photovoltaic parameters of the Ref, LCP, MCP, RCP CZTS thin‐film devices. *J_SC_* ^a^ is the integrated *J_SC_* from EQE.

CZTS Solar Cells	*J_SC_* [mA/cm^2^]	*J_SC_* ^a^ [mA/cm^2^]	*V_OC_* [mV]	*FF* [%]	*PCE* [%]	*n*	*J*_0_ [A/cm^2^]	*R_sh_* [Ω.cm^2^]	*R_s_* [Ω.cm^2^]	$E_{g}^{*}$ [eV]	*E_U_* [meV]	*V*_*OC*,*def*_ [V]
Ref	20.0	18.3	721	50.5	7.3	4.9	5.7×10^−5^	368.9	2.6	1.59	35	0.59
LCP	22.0	21.0	664	53.4	7.8	4.0	2.8×10^−5^	431.6	2.3	1.50	32	0.57
MCP	23.7	22.0	658	62.9	9.8	2.5	9.5×10^−7^	686.4	2.4	1.46	33	0.52
RCP	21.8	20.8	708	66.1	10.2	2.1	4.7×10^−8^	621.2	2.1	1.44	34	0.47

To gain deeper insight into the mechanism behind these performance improvements, the *J–V* curves were analyzed using a single‐diode model [33, 34], with detailed fitting procedures provided in Note S2. The extracted diode parameters from the illuminated *J–V* curve, including the ideality factor (*n*), shunt resistance (*R_SH_*), series resistance (*R_S_*), and saturation current density (*J_0_*) are summarized in Table 1. The Ref device exhibits a relatively low *R_SH_* of 368.9 Ω·cm^2^ and a high *R_S_* of 2.6 Ω·cm^2^, consistent with large resistive and shunt losses. Following device annealing and controlled cooling, *R_SH_* increases significantly (up to 686.4 Ω·cm^2^), while *R_S_* decreases to as low as 2.1 Ω·cm^2^, consistent with reduced resistive and leakage losses. The ideality factor (n) decreases from 4.9 in the ref device to 2.1 for the RCP device, while *J_0_* decreases dramatically from 5.7 × 10^−^^5^ to 4.7 × 10^−^^8^ A·cm^−^^2^ an improvement of nearly three orders of magnitude, which is lower than reported for the current world‐champion solution‐processed CZTS device (1.5 × 10^−7^ A·cm^−^^2^) [8]. In Table S4, we also extracted the diode parameters from the dark *J–V* curves. A similar Ref‐to‐RCP trend is observed, with *n* decreasing from 2.7 to 2.3 and decreasing *J_0_* from 1.3 × 10^−7^ to 1.8 × 10^−8^ A·cm^−^^2^, corresponding to an approximately sevenfold reduction. The reduced *n* and *J_0_* values for the RCP device are consistent with suppressed recombination losses [35], indicating improved diode characteristics after rapid cooling.

The markedly reduced *n* and *J_0_* values for the RCP device indicate strong suppression of recombination at the CdS/CZTS heterojunction [35]. These results demonstrate that rapid cooling effectively preserves the beneficial effects induced during annealing while simultaneously promoting improved heterojunction quality. Consequently, this cooling strategy leads to a significant enhancement in overall device performance, consistent with the earlier discussion. To verify the improved *J_SC_* due to cooling dynamics after device annealing, EQE measurements were performed. Figure 1d shows the EQE spectra and corresponding integrated *J_SC_* values for all devices. In the short‐wavelength region (350–450 nm), all cooling devices exhibit a modest increase in EQE, which may arise from changes in window‐layer optical transmission and/or carrier collection following device annealing, consistent with previous observations [16]. A more pronounced improvement is observed in the long‐wavelength region (750–900 nm), where the EQE response changes for all cooling devices. This change is attributed to Cd diffusion from the CdS buffer layer into the CZTS absorber (discussed in the next section) and changes in the degree of cation disorder during cooling dynamics after device annealing, which induces a slight bandgap narrowing and thereby improves long‐wavelength carrier generation, which is consistent with previous studies [12, 15, 16, 17, 18]. To support the interpretation of disorder‐related bandgap narrowing, we extracted the optical bandgap from the EQE spectra (Figure S11). A clear reduction in bandgap is observed when heat treatment (300°C for 12 min followed by rapid cooling) was performed after sulfurization. In this study, annealing was performed on the completed device stack, both Cd‐related intermixing near the CdS/CZTS heterojunction and changes in the CZTS cation‐disorder state may contribute to narrow the bandgap and improved *J_SC_*. The integrated *J_SC_* values calculated from the EQE spectra are systematically lower than those obtained from the *J–V* measurements; however, the mismatch remains below 10%, which is generally acceptable, and likely arises from differences in measurement conditions.

**FIGURE 2:**
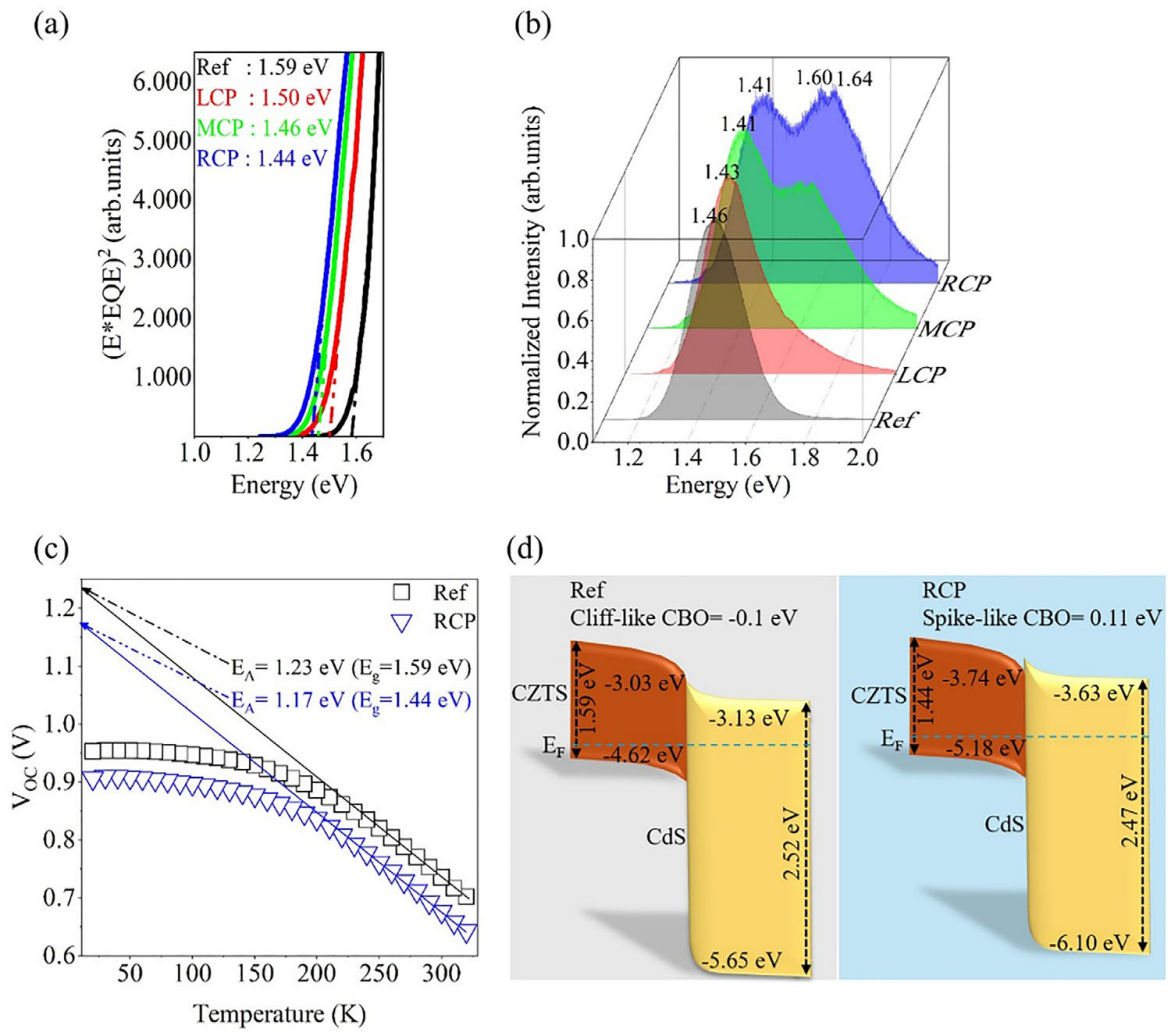
(a) Bandgap (*E_g_*) extraction using (EQE·E)^2^ versus photon energy plots. (b) Normalized room‐temperature PL spectra of the Ref, LCP, MCP, and RCP devices. (c) Temperature‐dependent *V_OC_* curves for ref and RCP devices, used to determine activation energy (*E_A_*) from the extrapolated *y*‐axis intercept at T = 0 K. (d) Schematic illustration of the band alignment at the heterojunction CZTS/CdS for ref and RCP devices.

Figure 2a shows bandgap values extracted from the EQE, as calculated in Note S3. The bandgap values gradually decreased from 1.59 eV for the Ref device to 1.50 eV for LCP, 1.46 eV for MCP, and 1.44 eV for RCP. These results confirm a gradual reduction in bandgap with different cooling processes, consistent with enhanced Cd incorporation into the CZTS lattice—most likely via substitution at Zn sites— as well as increased cation disorder [16, 17, 18]. Furthermore, the Urbach energy (*E_U_*) was extracted from the EQE spectra to evaluate the influence of cooling protocol after device annealing on band‐tail states. In Figure S12, *E_U_* decreased from 35 meV for the Ref device to 32 meV for the LCP device. In contrast, *E_U_* slightly increased to 32 meV for the LCP device and further to 34 meV for the RCP device. The nearly unchanged *E_U_* indicates that different cooling protocols after device annealing have virtually no significant effect on the band‐tail states.

Room‐temperature photoluminescence (RT‐PL) spectra are shown in Figure 2c. The PL peak shifts from 1.46 eV in the Ref device to 1.43 eV in the LCP device, and to ∼1.41 eV in both MCP and RCP devices. Furthermore, MCP and RCP devices exhibit two distinct PL features: a CZTS‐related peak (P1) at ∼1.41 eV and a higher‐energy peak (P2) at ∼1.60 eV associated with CdS [30, 36, 37]. The P2 peak near 1.60 eV is attributed to Cu^2^^+^‐related transitions within CdS [36]. Moreover, the non‐normalized room‐temperature PL spectra in Figure S13 show that the MCP and RCP devices have much lower PL intensity than the LCP and Ref devices. In general, higher band‐band transition PL intensity is frequently associated with high device efficiency [38]. However, in the present case, the highest PCE is obtained from devices exhibiting reduced CZTS absorber PL intensity.

To further elucidate the impact of cooling dynamics on device performance, the evolution of the *V_OC_* deficit (*V_OC, def_*) was analyzed. The *V_OC, def_* provides a direct comparison between the experimentally measured *V_OC_* and the maximum achievable (radiative) $V_{OC}^{SQ\;}$ defined by the Shockley–Queisser (SQ) model, expressed as follows: [39]

(1) $$V_{OC,def}=V_{OC}^{SQ}-\;V_{OC}^{exp}$$

where the radiative *V_OC_* can be approximated as a function of bandgap as follows

(2) $$V_{OC}^{SQ}=0.932\times E_{g}-0.167$$

Because *V_OC_* is fundamentally linked to the absorber bandgap (*E_g_*), *V_OC, def_* is a key parameter to distinguish whether variations in *V_OC_* arise primarily from bandgap changes or from improved defect passivation and charge recombination suppression. *V_OC, def_* was calculated using the EQE‐derived bandgaps of 1.59, 1.50, 1.46, and 1.44 eV for the Ref, LCP, MCP, and RCP devices, respectively. The Ref device exhibits a *V_OC, def_* of 0.59 V, which decreases progressively to 0.57 V for the LCP device, 0.52 V for the MCP device, and 0.47 V for the RCP device. This clear downward trend demonstrates that cooling rate after device annealing systematically reduces the *V_OC_* deficit, which indicates a substantial suppression of non‐radiative recombination pathways and an enhancement in interfacial quality and carrier collection [7, 15]. This improvement is consistent with the discussion of the electronic properties.

For the following analysis, the Ref device and the best‐performing device (RCP) were selected for further analysis. We measured temperature‐dependent current–voltage (*JV–T*) measurements at 20–320 K. This measurement is widely used to calculate the activation energy (*E_A_*) (in Note S5). In general, the difference between *E_A_* and *E_g_* provides insight into the CdS/CZTS heterojunction quality, where a smaller difference indicates a better‐quality junction, and vice versa. Figure 2c presents the extracted *E_A_* values for the Ref and RCP devices. The Ref device exhibits an *E_A_* of 1.23 eV, which is substantially lower than its *E_g_*, resulting in a large *E_g_ – E_A_* value of 0.36 eV (Table 2). This large deviation indicates that recombination is dominated by interface‐related defect states associated with a poor heterojunction. In contrast, the RCP device shows an *E_A_* of 1.17 eV and the small *E_g_ – E_A_* difference of 0.27 eV (in Table 2), indicating improved junction quality with suppressed interface recombination, consistent with the better performance of RCP device. Furthermore, capacitance–voltage (*C–V*) measurements were conducted and calculated in Note S6 to extract the charge density (*N_CV_*) and depletion region width (*W_d_*) of the Ref and RCP devices. As shown in Figure S15 and summarized in Table 2, the Ref device shows an *N_CV_* of 1.2 × 10^16^ cm^−^^3^ and corresponding *W_d_* of 386 nm. In contrast, the RCP device shows a higher *N_CV_* of 2.7 × 10^16^ cm^−^^3^, accompanied by a narrower *W_d_* of 140 nm. The increase in *N_CV_* observed after heat treatment of heterojunction or full device is consistent with prior reports [8, 10, 16, 25]. Furthermore, temperature‐dependent admittance spectroscopy (AS) was employed to probe electrically active defect states in the Ref and RCP devices. Figure S16a,b shows the admittance spectra of Ref and RCP devices. In Note S7, we calculated the activation energy from the Arrhenius plot shown in Figure S16c. The Ref device exhibits an activation energy of ∼222 meV, which lies within the range frequently reported for the main admittance feature in kesterite solar cells [40, 41]. In contrast, the RCP device shows a reduced activation energy of ∼136 meV, consistent with the commonly reported acceptor‐activation energy in kesterite absorbers (∼130–200 meV) [17, 40, 41, 42, 43, 44]. To gain more insight into defect‐related recombination and bulk changes after rapid cooling, we measured temperature‐dependent photoluminescence (PL), and the results will be discussed further below.

**TABLE 2:** Summary of parameters extracted from *JV(T)*, C–f, and C–V measurements. *E_A_* is the activation energy obtained from JV–T measurement, *E_a_* is the defect‐level activation energy derived from C–f measurements. *N_CV_* and *W_d_* denote the net space‐charge density and depletion‐region width, respectively, extracted from C–V measurement.

CZTS Solar Cells	*E_A_* [eV]	*E_g_ – E_A_* [eV]	*E_a_* [meV]	*N_CV_* [cm^−3^]	*W_d_* [nm]
Ref	1.23	0.36	222 ± 10	1.2×10^16^	386
RCP	1.17	0.27	136 ± 13	2.7×10^16^	140

To quantify the improvement of CdS/ CZTS heterojunction from the band alignment side, we utilized ultraviolet photoelectron spectroscopy (UPS) to determine the valence band maximum (VBM) and secondary electron cutoff (*E_cutoff_*) of the Ref and RCP devices. Band alignment at the CdS/ CZTS interface is typically classified as either cliff‐type or spike‐type. Figure S17 shows UPS results of the *E_cutoff_* and valence‐band onset (*E_onset_*). For the Ref device, the Fermi level (*E_F_*) positions relative to the VBM were determined to be 1.38 eV for CdS and 0.23 eV for CZTS. The RCP device shows *E_F_* values of 1.39 eV for CdS and 0.14 eV for CZTS. The shift of the CZTS Fermi level toward the VBM after RCP treatment indicates enhanced p‐type character (i.e., increased surface acceptor density) [8]. Figure 2d shows the band alignment of CZTS/CdS for the Ref and RCP devices, with *E_g_* extracted from EQE spectra. Quantitative evaluation of the band alignment yields CBO values of − 0.1 eV for the Ref device and + 0.11 eV for the RCP device as illustrated in Figure 2d. The negative CBO in the Ref device corresponds to a cliff‐like alignment, which promotes majority carrier recombination at the interface and limits device performance. In contrast, the RCP device exhibits a positive CBO (spike‐type alignment), approaching the optimal band alignment regime. This favorable interfacial energetics suppresses recombination, enhances charge carrier collection, and results in improved *FF* and increased *J_SC_* [7, 8]. Interestingly, this spike‐like band alignment of 0.11 eV achieved by device annealing in ambient air in this study is identical to that obtained by heterojunction treatments performed under oxygen‐rich atmospheres [8].

**FIGURE 3:**
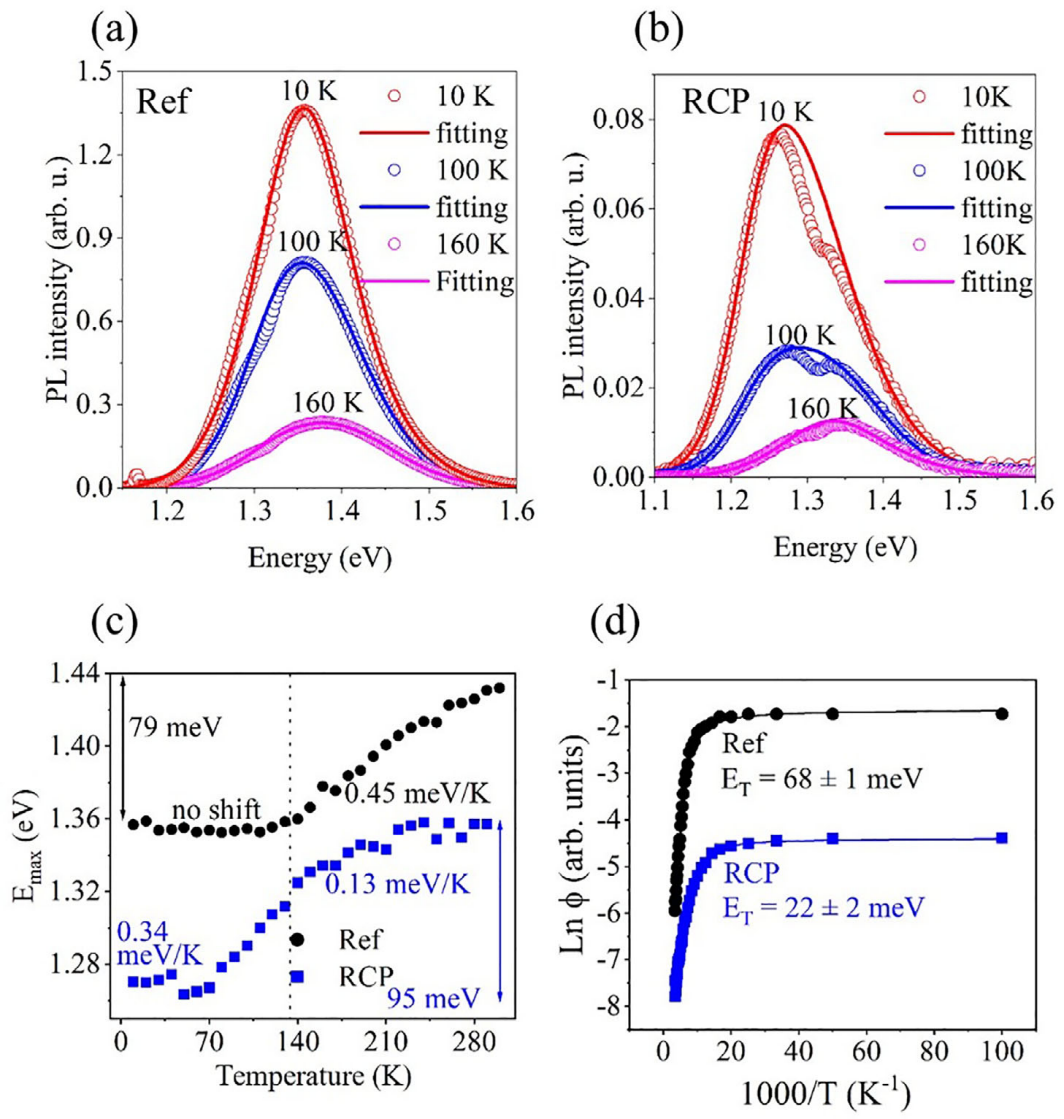
Fitting result of the Photoluminescence (PL) spectrum at T = 10, 100, and 160 K using an ADS function. (a) Ref device. (b) RCP device. (c) Temperature dependence of the PL peak position (*E_max_*). Arrhenius plot derived from the temperature‐dependent PL spectra, showing the extracted thermal activation energies (*E_A_*) (d) ref and RCP devices.

To expand the discussion of radiative recombination pathways and the defect landscape, we performed temperature‐dependent photoluminescence (PL) on Ref and RCP devices. PL was measured on completed device stacks (Mo/CZTS/CdS/i‐ZnO/ITO) under pulsed‐laser excitation. Figure 3a,b presents the fitting result of the PL spectra at 10, 100, and 160 K for Ref and RCP. In both cases, a single broad and asymmetric emission band is observed. In the Ref and RCP devices (Figure S18), a clear dip near 1.32 eV is seen due to water‐vapor absorption [45]. To quantify the line shape, the spectra were fitted with an asymmetric double sigmoidal (ADS) function, as shown in detail in Note S9. In the low‐temperature range (10–130 K), peak energy (*E_max_*) of the Ref remains nearly constant, whereas the RCP sample exhibits a blueshift with a rate of 0.34 meV/K. In the higher‐temperature region (140–300 K), *E_max_* continues to blueshift with rates of 0.45 and 0.13 meV/K for the Ref and RCP samples, respectively (Figure 3c). In the Ref sample, *E_max_* does not change much at low temperature, suggesting that only a limited number of recombination states are available. At higher temperatures, carriers gain enough energy to move into closer donor–acceptor pairs, causing *E_max_* to blueshift. In the RCP sample, *E_max_* starts to increase already at 10 K. The total *E_max_* shift (76–95 meV) is of the same order as the Coulomb energy expected for recombination between closely spaced defect pairs (r ≈ 1–2 nm) in CZTS [46]. Overall, recombination in the RCP sample occurs at more closely separated pairs, indicating a stronger band bending, enabling recombination to happen at shallower defect levels. Subsequently, PL quenching in both samples is governed by a single thermally activated process, as shown in Figure 3d. The temperature dependence of ϕ *(T)* as a function of *1000/T* was fitted using the following expression [47]:

(3) $$\phi \;\left(T\right)=\phi_{0}/\left[1+A_{1}T^{3/2}+A_{2}T^{3/2}exp\left(-E_{T}/kT\right)\right]\;$$

Here, ϕ denotes the integrated PL intensity, *A_1_* and *A_2_* are rate parameters, and *E_T_* is the thermal activation energy. The extracted activation energies are 68 and 22 meV for the Ref and RCP samples, respectively, and are attributed to the ionization of shallow acceptor defects. The results suggest that the bulk defects are not significantly changed in the RCP device. Therefore, the improved device performance mainly comes from the modified heterojunction, consistent with our previous discussion.

### 2.2 Effects of Rapid Cooling on CdS/CZTS Heterojunction and Back Interfaces

**FIGURE 4:**
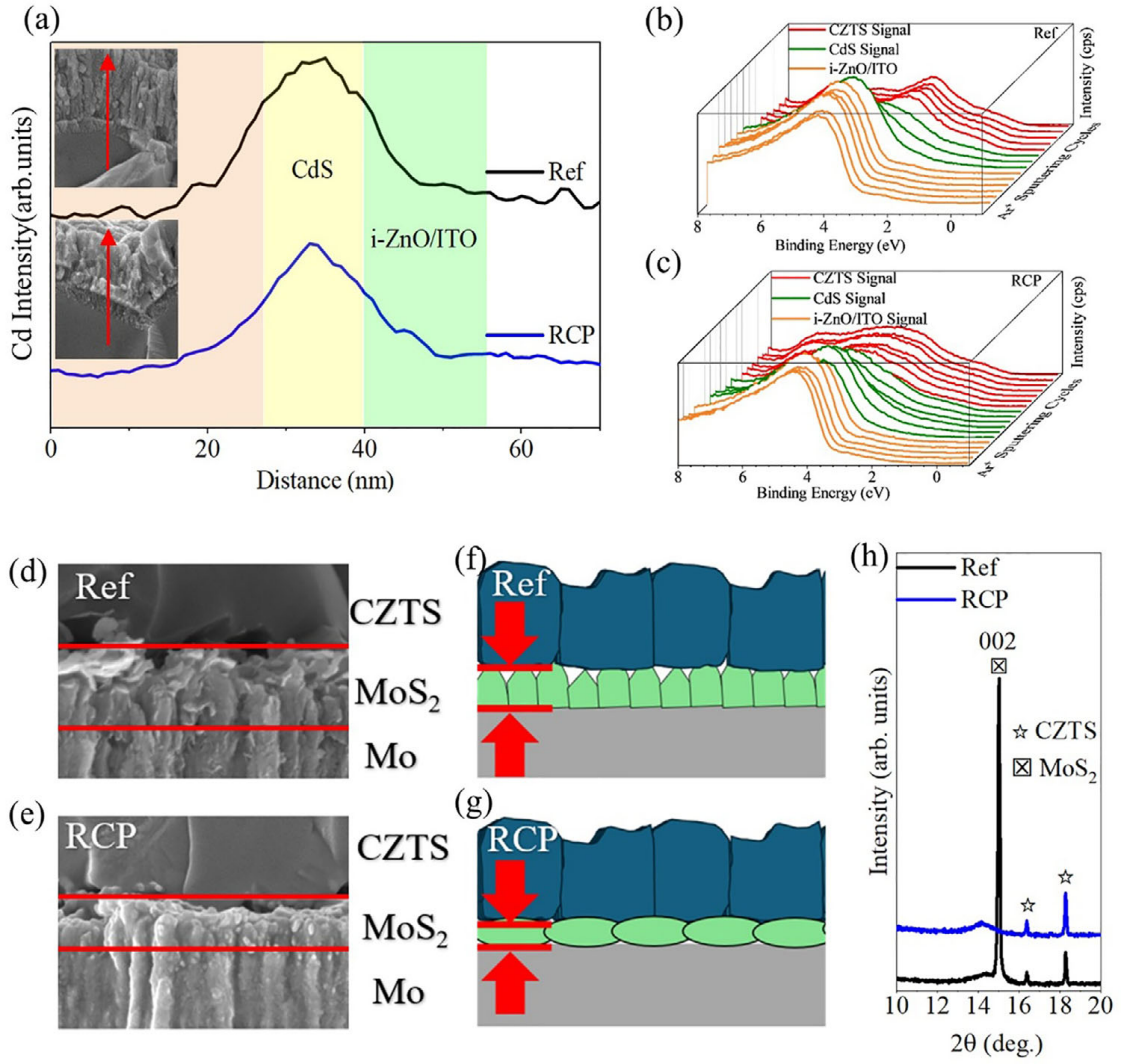
Cross‐sectional high‐resolution scanning electron microscopy (HR‐SEM) images of CZTS/CdS/i‐ZnO/ITO devices for the (a) Energy‐dispersive X‐ray spectroscopy (EDS) Cd line profiles across the CdS/CZTS heterojunction for the ref (black) and RCP (blue) devices, illustrating enhanced Cd in‐diffusion after device annealing with rapid cooling. (b, c) UPS depth‐profiling spectra of the CZTS/CdS/i‐ZnO/ITO stack for the (b) Ref and (c) RCP devices in the 0–8 eV binding‐energy range; photoemission intensity is plotted versus binding energy for successive etch levels, with binding energy referenced to the Fermi level. (d, e) Cross‐sectional HR‐SEM images of the Mo/MoS_2_/CZTS back contact for the (d) ref and (e) RCP devices. (f, g) Schematic illustrations depicting the modification of the MoS_2_ interfacial layer induced by rapid cooling following device annealing. (h) XRD results of the ref and RCP devices.

As discussed above, device annealing leads to an increase in *J_SC_* accompanied by a slight reduction in the apparent CZTS bandgap extracted from EQE analysis. This bandgap narrowing is attributed to cation disorder and Cd interdiffusion from the CdS buffer layer into the near‐interface CZTS region. To approve the interdiffusion element during device annealing, we examined the Ref and RCP devices with cross‐sectional high‐resolution scanning electron microscopy (HR‐SEM in Figure 6a–b) and energy dispersive X‐ray spectroscopy (EDX) line profiling across the heterojunction. Figure 4a observes a broader Cd distribution after device annealing (RCP device). This broadened CdS‐related response is also evident in the UPS depth profile as illustrated in Figure 4b,c, which is consistent with previous study [15, 16, 18, 24]. The broadening of the CdS signal after annealing suggests Cd diffusion/incorporation into the near‐surface CZTS region (e.g., occupation of cation sites), accompanied by modifications of the near‐interface electronic structure (bandgap and band alignment). These changes are consistent with reduced interfacial recombination, improved carrier collection, and a more favorable junction band alignment [8, 10, 14, 16, 18, 24, 48].

Beyond the front heterojunction, the cooling dynamics after device annealing also markedly influences the Mo/CZTS back contact. The Ref device exhibits an interfacial gap at the Mo/CZTS interface, which is consistent with the formation of a MoS_2_ secondary phase during high‐temperature sulfurization. Such an interfacial layer is known to increase series resistance, limit the *FF*, and ultimately degrade device performance [16, 49, 50, 51, 52]. In this context, Yang et al. showed that thermal treatment of the Mo back contact before precursor deposition can suppress excessive MoS_2_ formation while promoting beneficial Na diffusion from soda‐lime glass (SLG) into the Mo and CZTS absorber, thereby reducing recombination losses and improving performance; increasing the Mo annealing temperature to 550°C decreased the MoS_2_ thickness from 179 to 114 nm and increased device efficiency [49]. Similar interfacial‐layer effects are well known in chalcopyrite devices, where the MoSe_2_ thickness at the CIGS/Mo interface governs the trade‐off between contact resistance and adhesion [51]. Overall, controlling the thickness and continuity of the MoX_2_ interfacial layer (MoS_2_ in CZTS, MoSe_2_ in CIGS) is crucial for efficient charge extraction without compromising mechanical integrity. To evaluate the impact of our cooling protocol on the CZTS/Mo back contact, we examined the interface by HR‐SEM. Cross‐sectional HR‐SEM reveals a distinct MoS_2_‐related interfacial region and gap in the Ref device (Figure 4d), which can act as a physical barrier for hole extraction and contribute to *FF* losses. In contrast, the RCP device shows a more compact and continuous Mo/CZTS interface (Figure 4e), suggesting that rapid cooling “freezes in” the annealing‐modified back‐contact configuration, effectively densifying the interfacial region and minimizing gap formation. To further assess how RCP influences the back‐contact interfacial phase, we compared the XRD patterns of the Ref and RCP devices (Figure 4h). The Ref device exhibits a pronounced MoS_2_ diffraction peak at 15.1°, assigned to the (002) plane, whereas this feature is strongly suppressed in the RCP device. This attenuation could be a modified MoS_2_ after rapid cooling following device annealing, as schematically illustrated in Figure 4f,g. To further assess the back‐contact properties, temperature‐dependent *J–V* measurements were compared for the Ref and RCP devices (Figure S22). With decreasing temperature, the Ref device shows a more pronounced roll‐over behavior, particularly at ∼100 K, whereas the RCP device exhibits a weaker roll‐over effect. Because low‐temperature roll‐over can be associated with a carrier‐extraction barrier, this difference is consistent with improved carrier extraction after rapid cooling [53]. Together with the HR‐SEM and XRD observations, these results provide complementary evidence consistent with modified back‐contact properties.

To examine whether rapid cooling after annealing can redistribute the MoS_2_ interfacial layer, we carried out a control experiment on Mo‐coated soda–lime glass (Mo/SLG) using the same conditions as in Figure S20. After sulfurization, the samples were either left untreated (“without annealing, in Figure S20a”) or subjected to a post‐anneal at 300°C for 12 min followed by rapid cooling (“with annealing + RCP, in Figure S20b”). Cross‐sectional SEM shows that the MoS_2_ layer is thicker in the sample without annealing, whereas the annealed + RCP sample exhibits a reduced/denser MoS_2_ interfacial region. Consistently, top‐view SEM indicates a higher surface distribution of MoS_2_ features for the sample without annealing compared with the annealed + RCP sample. These results support our interpretation that rapid cooling after annealing promotes modification of MoS_2_, thereby mitigating excessive MoS_2_ formation at the back contact. Notably, while back‐contact optimization in kesterite devices is typically achieved through Mo deposition control or high‐temperature treatments [49, 50, 51, 52], the present results indicate that cooling‐rate control after device annealing offers an additional and practical route to modulate MoS_2_‐related interfacial evolution. To our knowledge, such cooling‐induced modification of the Mo/CZTS back contact has not been previously reported in kesterite device‐annealing studies.

### 2.3 Stability Measurement of the RCP Device

**FIGURE 5:**
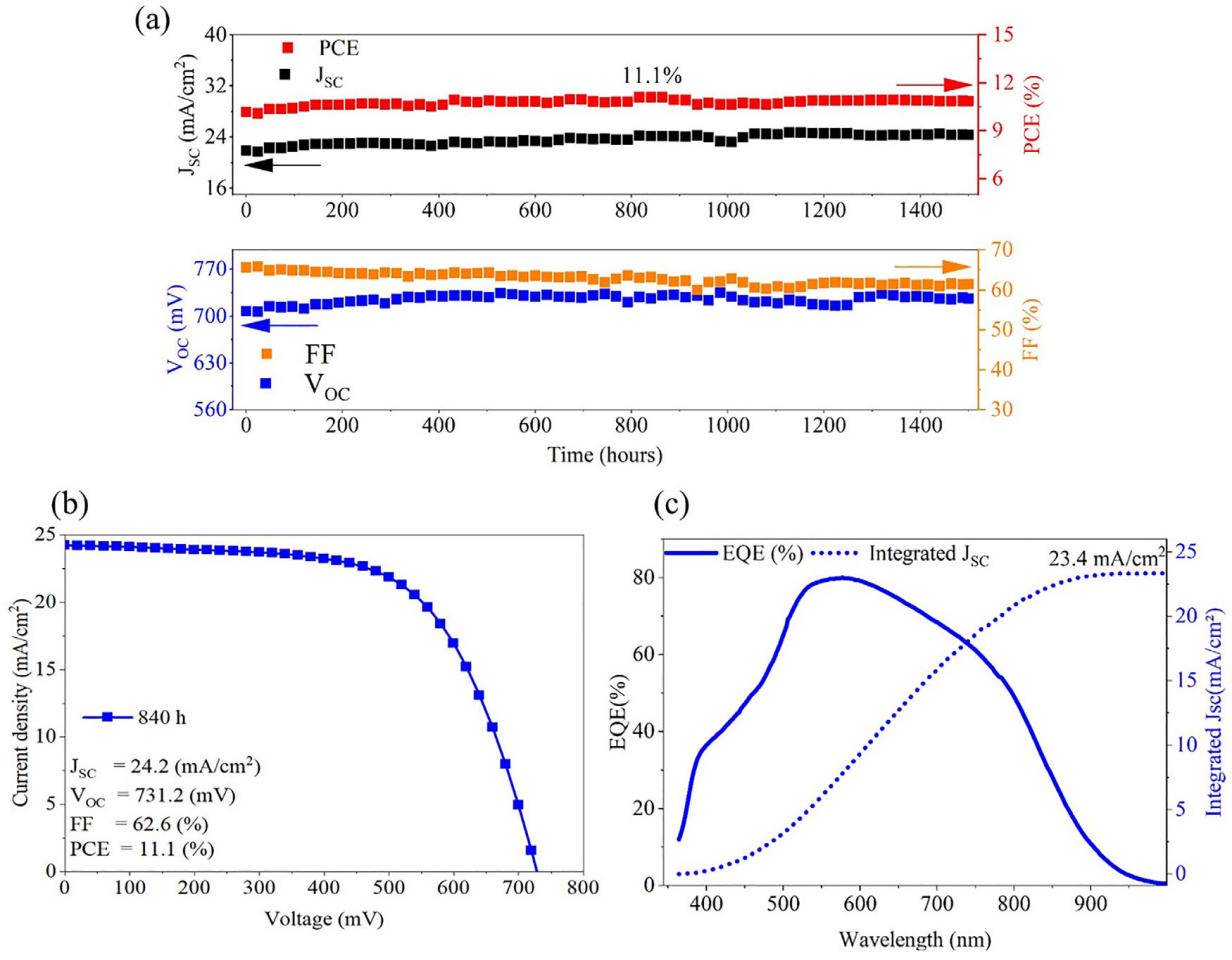
Long‐term stability of the RCP CZTS solar cell stored at 85°C in an oven. (a) Evolution of the photovoltaic parameters (*J_SC_*, *V_OC_*, *FF*, and PCE) as a function of storage time. (b–c) *J–V*, EQE, and integrated *J_SC_* curves measured for after approximately 840 h.

To assess the long‐term performance stability of the RCP device, extended stability measurements were conducted under accelerated dark aging in ambient air at 85°C without encapsulation. The device was stored in an oven for 1500 h and periodically removed for *J–V* characterization at room temperature. As shown in Figure 5a, the photovoltaic parameters exhibit stable and even improving trends over time. Interestingly, *J_SC_*, *V_OC_*, and PCE gradually increase during the aging process, while the *FF* shows a moderate decrease of approximately 5%, from 66.1% (fresh device) to 62.8% for 1500 h, which is attributed to gradual erosion of the Au front contact over time, as evidenced by the formation of surface scratches observed in Figure S23 after frequent handling during prolonged measurements. Despite this small *FF* reduction, the overall device efficiency continues to improve. Remarkably, after ∼800 h of aging at 85°C, the RCP device achieves a *PCE* of 11.1%, with a *J_SC_* of 24.2 mA cm^−^^2^, a *V_OC_* of 731.2 mV, and an *FF* of 62.6%, as presented in Figure 5b. This performance enhancement is primarily driven by a simultaneous increase in *J_SC_* of ∼2.4 mA cm^−^^2^ and *V_OC_* of ∼24 mV, achieved without the use of encapsulation and anti‐reflection coatings.

Furthermore, the champion device was benchmarked against the highest‐performing pure‐sulfide CZTS solar cells reported to date by compiling key photovoltaic metrics and evaluating the voltage‐deficit indicator *V_OC_*/$V_{OC}^{SQ\;}$ (Table 3). The corresponding $V_{OC}^{SQ\;}$ values were calculated using Equation (1), and entries marked with “*” were independently recalculated for consistency. Notably, the RCP device delivers a *V_OC_*/$V_{OC}^{SQ\;}$ of 62.4%, representing the highest among the listed solution‐processed CZTS devices and approaching the level of state‐of‐the‐art sputtered CZTS devices. This reduced voltage deficit is consistent with suppressed interfacial recombination enabled by rapid cooling after device annealing. Moreover, the favorable efficiency evolution under 85°C aging further suggests that cooling‐rate engineering can simultaneously improve junction quality and operational robustness.

**TABLE 3:** Summary of the photovoltaic performance parameters of the highest‐performing CZTS solar cells reported in the literature and in this work. Values marked with “*” indicate that $V_{OC}^{SQ\;}$ values were independently calculated using Equation (1) [39].

Absorber	Fabrication Process	*J_SC_* [mA/cm^2^]	*V_OC_* [mV]	*FF* [%]	*PCE* [%]	*E_g_* [eV]	*V_OC_ /* $\;V_{OC}^{SQ\;}$ [%]	Refs.
CZTS	Monograin	23.6	735	67.7	11.7	1.54	57.6*	[54]
CZTS	Sputtering	21.7	783	69.0	11.8	1.53	62.1*	[7]
CZTS	Solution	25.2	728	68.3	11.9	1.50	58.3	[8]
CZTS	Solution	24.2	731	62.6	11.1	1.44	62.4*	This study

### 2.4 Indoor Photovoltaics Performance of the RCP Device

**FIGURE 6:**
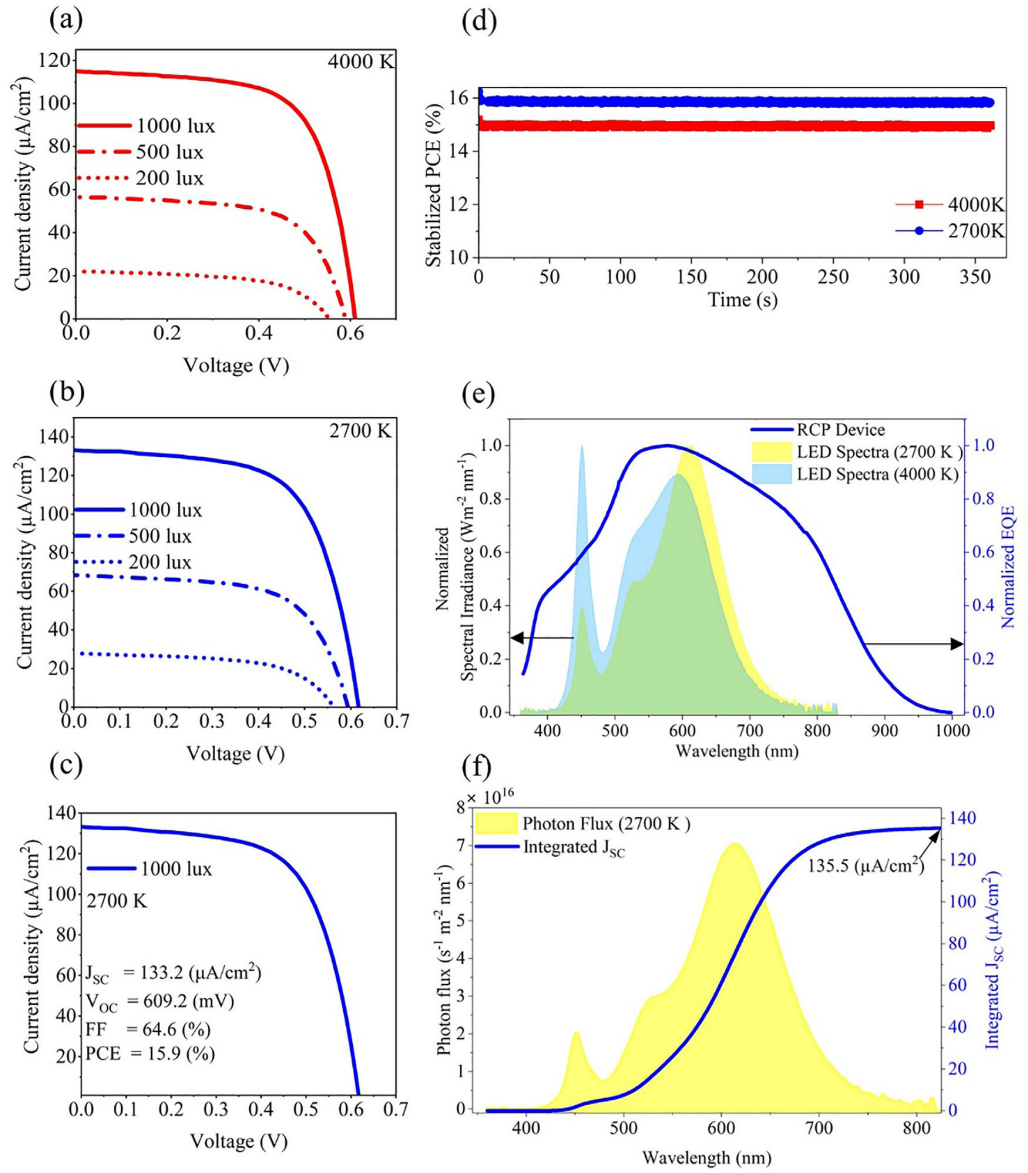
(a–b) *J–V* curve of the champion device, measured under 4000 and 2700 K LED illumination at 1000, 500, and 200 lux. (c) The *J–V* curve of a champion device with the highest i‐PCE of 15.9% under 2700 K at 1000 lux. (d) Stable power output for the champion device obtained via the maximum power point (MPP) under 4000 and 2700 K at 1000 lux. (e) Normalized emission spectrum of 4000 and 2700 K, compared with the EQE spectra of the champion device. (f) Photon flux density spectrum of 2700 K at 1000 lux and the integrated *J_SC_* for the champion device.

To evaluate the indoor photovoltaic performance, the RCP device was measured under 2700 and 4000 K LED illumination at 1000, 500, and 200 lux, following the IEC TS 62607‐7‐2:2023 guidelines for standardized indoor PV characterization [55]. Figure 6a,b shows the corresponding *J–V* curves under different illumination intensities. Under 1000 lux, the RCP device delivered an indoor PCE of 15.9% under 2700 K LED illumination, with a FF of 64.8%, *J_SC_* of 133.2 µA cm^−^^2^, and *V_OC_* of 609.9 mV. Under 4000 K illumination, the device achieved a PCE of 15.2%, with a FF of 66.6%, *J_SC_* of 115.2 µA cm^−^^2^, and *V_OC_* of 609.9 mV, as summarized in Table S5. To the best of our knowledge, the 15.9% efficiency represents the highest reported indoor PCE for pure‐sulfide CZTS solar cells under indoor testing conditions at 1000 lux. This value exceeds the previously reported 15.1% efficiency of alkali‐doped CZTS devices measured under 3000 K at 9586 lux [5]. Subsequently, we measured the device with decreasing illumination intensity from 1000 to 200 lux for both LED spectra, resulting the decreased indoor PCE, mainly due to the reduced amount of photon under weaker illumination [56]. Notably, the RCP device maintained better performance under 2700 K illumination, with the PCE decreasing only from 15.9% to 13.9%, whereas a larger reduction of approximately 24% was observed under 4000 K illumination. The reliability of the *J–V* measurements was verified using stable power output (SPO) measurements under 2700 and 4000 K LED illumination at 1000 for 350 s. As shown in Figure 6d, the device exhibited stable power output under both 2700 and 4000 K LED illumination. Finally, the reliability of the indoor photovoltaic measurements was verified by comparing the *J_SC_* obtained from *J–V* measurements with the integrated *J_SC_* calculated from the EQE spectrum and the 2700 K LED photon flux spectrum. The integrated *J_SC_* from EQE was 135.5 µA cm^−^^2^, which closely agrees with the *J_SC_* of 133.2 µA cm^−^^2^ obtained from the measurement under 1000 lux 2700 K illumination, corresponding to a difference of only ∼1.7%. To verify the reproducibility of the 1000 lux light source, the illuminance and power density were measured 17 times under identical conditions. The resulting mean values were 1004.0 ± 0.9 lux and 3265.2 ± 6.1 mW m^−^^2^, where the uncertainties represent one standard deviation. The corresponding relative standard deviations are approximately 0.09% and 0.19%, respectively, demonstrating high stability of the illumination source. The individual measurements are provided in Table S6. These results establish cooling dynamics after device annealing as a key yet underexplored parameter in CZTS device engineering and provide a simple, scalable route toward efficient and durable kesterite photovoltaics for both outdoor and indoor applications.

## 3 Conclusions

In summary, we demonstrate that controlling cooling dynamics after device annealing is an effective strategy to enhance both efficiency and operational stability of wide‐bandgap CZTS solar cells for outdoor and indoor conditions, while providing insight into the underlying mechanisms. Rapid cooling after device annealing changed both the heterojunction and back‐contact interfaces. At the CdS/CZTS interface, RCP devices suppress non‐radiative recombination by shifting the band alignment from a cliff‐like to a more favorable spike‐type configuration, consistent with the reduced *V_OC_* deficit and the nearly three‐order‐of‐magnitude decrease in *J_0_*. These improvements aligned with temperature‐dependent PL result that the bulk defects in the RCP device did not change significantly. This indicates that the main improvement comes from the modified heterojunction. At the back contact, RCP devices mitigate interfacial gap formation and reconfigure the interfacial MoS_2_ layer, enabling more efficient hole extraction and potentially reducing back‐contact losses. As a result, RCP devices achieve a champion PCE of 11.1% after stability measurement for ∼1500 h of aging at 85°C, and the corresponding *V_OC_* / *V_OC, SQ_* of 62.4% is among the highest reported for solution‐processed pure‐sulfide CZTS solar cells. Notably, the RCP device delivers an indoor PCE of 15.9% under 2700 K LED illumination, with a FF of 64.8%, *J_SC_* of 133.2 µA cm^−^^2^, and *V_OC_* of 609.9 mV. To the best of our knowledge, the 15.9% efficiency represents the highest reported indoor PCE for kesterite solar cells under indoor testing conditions at 1000 lux. These findings establish cooling dynamics after device annealing as a key yet underexplored parameter in CZTS device engineering and provide a simple, scalable route toward efficient and durable kesterite photovoltaics for both outdoor and indoor applications.

## 4 Experimental Section

### 4.1 Preparation of Precursor Solution and Film of CZTS

The CZTS solution was prepared by dissolving 40.7 mmol (3.098 g) of thiourea (TU), 9.35 mmol (0.925 g) of CuCl, 5 mmol (1.128 g) of SnCl_2_.2H_2_O, and 6 mmol (0.817 g) of ZnCl_2_, into 10 mL of dimethyl sulfoxide (DMSO) in a single vial. The molar ratios were adjusted to yield Cu‐poor and Zn‐rich compositions, consistent with our previous study [12]. The CZTS solution was stirred at ∼80°C until complete dissolution, resulting in a pale‐yellow solution. Prior to deposition, the CZTS solution was filtered through a 0.45 µm disposable syringe filter. CZTS solution was deposited on molybdenum‐coated soda‐lime glass (Mo/SLG, 5 × 5 cm^2^) by spin coating. Before deposition, the Mo/SLG substrates were cleaned by Hellmanex III diluted in ultrapure water (∼18.2 MΩ cm) at a 1:10 ratio, followed by ultrasonication at ∼30°C for 10 min, drying with high‐pressure nitrogen gas, and short annealing step at 350°C for ∼1 min. The CZTS solution was spin‐coated onto the cleaned Mo/SLG substrates for 10 cycles (yielding a total thickness ∼1.2 µm) at 3000 rpm for 30 s per cycle. Each deposition cycle was followed by annealing at 350°C for ∼2 min and cooling for ∼6 min. Notably, the CZTS precursor films were prepared entirely under ambient air. Subsequently, the CZTS precursor films were cut into 4 pieces (2.5 × 2.5 cm^2^). The resulting CZTS precursor films were sulfurized in a graphite box containing 300 mg of sulfur, using an OTF‐1200X tube furnace, following the multi‐stage temperature profile illustrated in Figure S1. Sulfurization was conducted under an argon atmosphere at an initial pressure of 600 Torr at room temperature.

### 4.2 Device Fabrication

After sulfurization, the CZTS thin films were immersed in ultrapure water (∼18.2 MΩ cm) for ∼2 min and then transferred to a beaker maintained at ∼60°C for chemical bath deposition (CBD) of the buffer layer. The CBD solution consisted of 0.003 M cadmium acetate (CdAc_2_), 0.1 M thiourea, and 3.85 M aqueous ammonia (NH_4_OH). CBD was conducted under continuous stirring for 8 min, resulting in a CdS buffer layer with a thickness of ∼40–60 nm. Then, ∼50 nm of i‐ZnO and ∼250 nm of indium tin oxide (ITO) were deposited by radio frequency magnetron sputtering. Finally, gold top contact grids (thickness of 100 nm) were deposited by sputtering system through shadow masks. The device area was defined by mechanical scribing, yielding a total active area of approximately 0.22–0.23 cm^2^. Device annealing was performed prior to Au deposition under ambient air on a hotplate, using a graphite box, for 12 min at temperatures of 150, 200, 250, 300, and 350°C. Based on the optimal annealing temperature, the devices were then subjected to different post‐annealing cooling protocols— rapid (1 min), moderate (4 min), and slow (60 min)—as illustrated in Figure 1a2.

### 4.3 Characterization Techniques

The structural, morphological, and compositional properties of the sulfurized CZTS absorber layers were systematically characterized. Morphology was examined by high‐resolution scanning electron microscopy (HR‐SEM; Zeiss Ultra 55) equipped with a backscattered‐electron detector. Elemental composition was determined by energy‐dispersive X‐ray spectroscopy (EDX; Bruker Esprit 1.82) using an XFlash 3001 detector. Raman spectra were recorded at room temperature using a HORIBA LabRAM 800HR micro‐Raman spectrometer with a 532 nm Nd:YAG laser excitation; the scattered light was analysed with an 1800 lines mm^−1^ grating and a Si CCD detector. The crystal structure was characterized by X‐ray diffraction (XRD; Rigaku Ultima IV) with a 9 kW rotating Cu anode (λ = 0.154 nm; 40 kV, 40 mA) in Bragg–Brentano geometry (2θ = 10–70°, step size 0.02°) using a D/teX Ultra silicon strip detector. Subsequently, for the CZTS device, XRD measurements were also performed using an Automated Multipurpose Powder X‐ray Diffractometer XRDynamic 500 by Anton Paar equipped with a Primux 3000 sealed‐tube Cu X‐ray source and a Pixos 2000 solid‐state hybrid pixel detector. Surface topography was measured by atomic force microscopy (AFM) using a Bruker MultiMode 8. Ultraviolet photoelectron spectroscopy (UPS) was carried out on an Axis Ultra DLD spectrometer (Kratos Analytical) using the He(I) resonance line (*h*ν  =  21.21 eV). Temperature‐dependent photoluminescence (PL) measurements were performed by mounting the CZTS solar cell on the cold finger of a closed‐cycle helium cryostat (Janis CCS‐150). The sample temperature was from 8 to 300 K and regulated using a LakeShore Model 335 temperature controller. PL excitation was provided by the second harmonic (532 nm) of a pulsed Q‐switched Nd:YAG laser (CryLaS FQSS266‐Q2) operating at 10 kHz, with a pulse width <1 ns and a pulse energy of 1.1 µJ (corresponding to a maximum average power of 7.5 mW). The excitation density was systematically tuned with neutral‐density filters, while maintaining a laser spot diameter of ∼1 mm on the device to ensure uniform illumination and minimize local heating effects. Device performance was evaluated by measuring current density–voltage (J–V) characteristics under AM 1.5G illumination (100 mW cm^−2^) using a Newport Oriel Class A 91195A solar simulator calibrated with a Si reference photodiode. External quantum efficiency (EQE) spectra were recorded using a commercial system (Sciencetech PTS‐2‐IQE, Canada). Capacitance–frequency, impedance spectroscopy, capacitance–voltage, and temperature‐dependent J–V measurements were carried out using a Wayne Kerr 6500B impedance analyzer, a closed‐cycle He cryostat (Janis), and a Keithley 2400 source meter.

### 4.4 Indoor Photovoltaic Measurements

Indoor photovoltaic and stable power output measurements were conducted under illumination from a Philips HUE WLED bulb set to color temperatures of 4000 and 2700 K. The illumination intensity was measured using a BTS256‐WF spectral light meter (GigahertzOptik). At 1000 lux, the WLED source provided optical power densities of approximately 0.31 mW cm^−^^2^ at 4000 K and 0.33 mW cm^−^^2^ at 2700 K at the device surface. All indoor measurements were carried out in a black box under ambient conditions. Calibrated spectral irradiance data at 1000 lux for both WLED spectra were collected using a spectroradiometer and converted to photon flux density for EQE‐based integrated current calculations.

## Author Contributions

A. Nasyori conceived and designed the study, led device fabrication, and developed measurement protocols. I. Mengü performed and analyzed photoluminescence measurements. M. Danilson carried out and analyzed UPS measurements, including CF and CV analysis. M. Pilvet contributed to window‐layer processing. J. Krustok assisted with J–V and PL data analysis. V. Mikli performed SEM characterization. R. Josepson and A. Nasyori performed and analyzed *JV–T*, *C–V*, and *C‐f* measurements. R. Kaupmees and A. Nasyori conducted AFM measurements. A. Akhil and P. Vivo assisted Indoor PV measurements and analysis. S. S. Hadke assisted with Urbach Energy and analysis. T. Unold assisted with electrical and optical analysis. Y. Gong assisted with data analysis. A. C. Galca performed XRD measurements. L. H. Wong assisted with data analysis, M. Grossberg‐Kuusk contributed to PL analysis. M. Kauk‐Kuusik coordinated overall data analysis. The manuscript was primarily written by A. Nasyori and I. Mengü (Temperature Dependent PL section) and revised by A. Nasyori, J. Krustok, S. S. Hadke, T. Unold, M. Grossberg‐Kuusk, and M. Kauk‐Kuusik. All authors reviewed the manuscript.

## Conflicts of Interest

The authors declare no conflicts of interest.

## Supporting information




**Supporting File**: advs77574‐sup‐0001‐SuppMat.pdf.

## Data Availability

The data that support the findings of this study are available from the corresponding author upon reasonable request.
