# Supplementary material for: Rapid Cooling After Device Annealing Suppresses Interface Losses in Cu2ZnSnS4 Solar Cells Enabling High Efficiency and Stability
Source: Adv Sci (Weinh). 2026 Sep 6:e77574. Online ahead of print. doi: 10.1002/advs.77574 (PMC13547715; doi:10.1002/advs.77574)
Supplement: Supplementary file 1 [file ADVS-9999-e77574-s001.pdf]

Supplementary Information

Rapid Cooling after Device Annealing Suppresses Interface Losses in Cu_2ZnSnS_4 Solar Cells Enabling High Efficiency and Stability

Achmad Nasyori,[1*] Mati Danilson,[1] Idil Mengü,[1] Maris Pilvet,[1] Jüri Krustok,[1] Reelika Kaupmees,[1] Valdek Mikli,[1] Raavo Josepson,[2] Yuancai Gong,[3,4] Akhil Alexander,[5] Shreyash S Hadke,[6] Aurelian Catalin Galca,[7,8] Paola Vivo,[5] Lydia Helena Wong,[9] Thomas Unold,[10] Maarja Grossberg-Kuusk[1] and Marit Kauk-Kuusik[1*]

[1] Department of Materials and Environmental Technology, Tallinn University of Technology, Ehitajate tee 5, 19086 Tallinn, Estonia.
[2] Division of Physics, Tallinn University of Technology, Ehitajate tee 5, 19086 Tallinn, Estonia.
[3] Photovoltaic Lab, Micro and Nano Technologies Group, Electronic Engineering Department, Barcelona East School of Engineering, Universitat Politècnica de Catalunya, Barcelona, Spain
[4] Barcelona Research Center for Multiscale Science and Engineering, Universitat Politècnica de Catalunya, Barcelona, Spain
[5] Hybrid Solar Cells, Faculty of Engineering and Natural Sciences, Tampere University, Tampere FI-33014, Finland
[6] Department of Materials Science and Engineering, Northwestern University, Evanston, IL, USA
[7] Laboratory of Complex Heterostructures and Multifunctional Materials (HeCoMat), National Institute of Materials Physics, Atomistilor 405A, 077125 Magurele, Ilfov, Romania.
[8] International Centre for Advanced Training and Research in Physics, Atomistilor 409, 077125 Magurele, Ilfov, Romania.
[9] School of Materials Science and Engineering, Nanyang Technological University, Singapore 639798, Singapore.
[10] Department Structure and Dynamics of Energy Materials, Helmholtz-Zentrum Berlin für Materialien und Energie, Lise-Meitner Campus, Hahn-Meitner-Platz 1, 14109 Berlin, Germany

Corresponding Author(s): Achmad Nasyori and Marit Kauk-Kuusik. Department of Materials and Environmental Technology, Tallinn University of Technology, Ehitajate tee 5, 19086 Tallinn, Estonia. Email: achmad.nasyori@taltech.ee and marit.kauk-kuusik@taltech.ee

Supplementary Note S1: Effect of temperature profile of sulfurization on CZTS thin films

To improve bulk quality, we employ a multi-stage sulfurization procedure (Figure S1) and test four distinct sulfurization temperature profiles. The sulfurization temperature of 650 °C with a dwell time of 20 min was identified as optimal for promoting CZTS crystallization under the present experimental conditions.[1] The multistage sulfurization approach was therefore designed to improve crystal growth kinetics, with the aim of larger grain formation at the absorber surface and enhancing device performance.

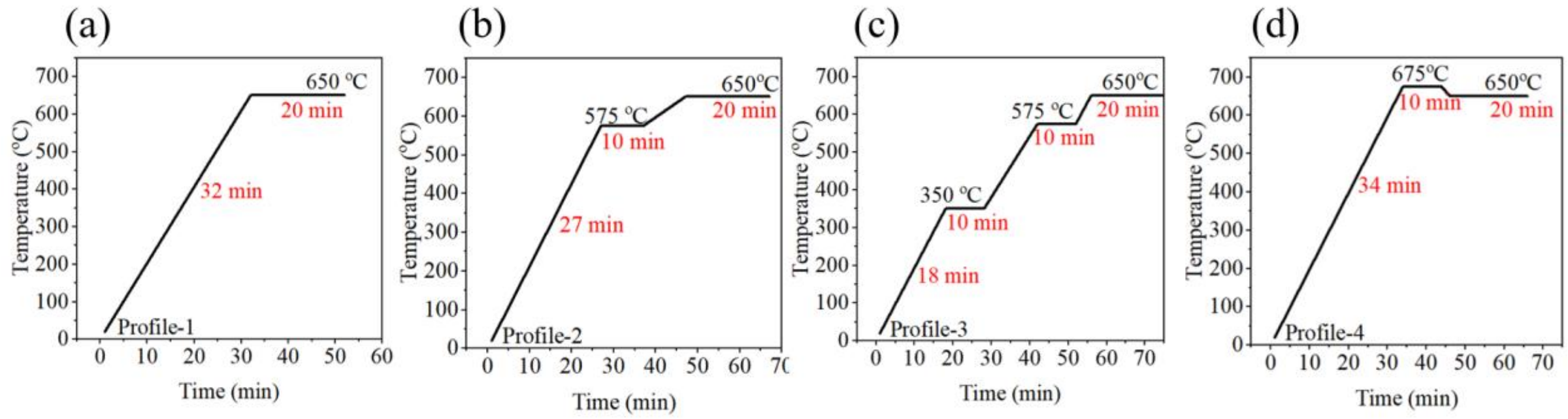

Figure S1. Schematic of the sulfurization temperature profiles for the different multi-stage processes

The grain growth behavior under different sulfurization profiles was investigated using scanning electron microscopy (SEM), and the corresponding top-view and cross-sectional images are presented in Figure 2. All sulfurization profiles produced compact and well-connected surface morphologies without observable pinholes, indicating dense absorber layers suitable for high-efficiency CZTS solar cell applications. As shown in Figure S2a1–d1, the Profile-1 process resulted in predominantly small grains, whereas Profile-3 and Profile-4 led to moderately enlarged grain sizes. In contrast, the Profile-2 sulfurization profile exhibited the most pronounced grain coalescence, yielding the largest average surface grains among the four conditions. The high density of small grains observed for Profile-1 is expected to increase grain boundary density and may therefore promote bulk recombination. Consequently, the larger grains obtained under Profile-2 condition are more favorable for efficient charge transport and are expected to benefit device performance. Cross-sectional SEM images (Figure S2a2–d2) show vertically oriented columnar grains typical of CZTS films. A thin interfacial gap between the Mo back contact layer and the CZTS absorber is visible for all samples, which may hinder charge extraction at the back contact. This observation suggests that further optimization of nucleation behavior or sulfurization initiation could enhance interfacial adhesion. Quantitative analysis of the top-view SEM images (Figure S2a3–d3) reveals average lateral grain sizes of 0.37 μm for Profile-1, 0.74 μm for Profile-2, 0.51 μm for Profile-3 and 0.67 μm for Profile-4.

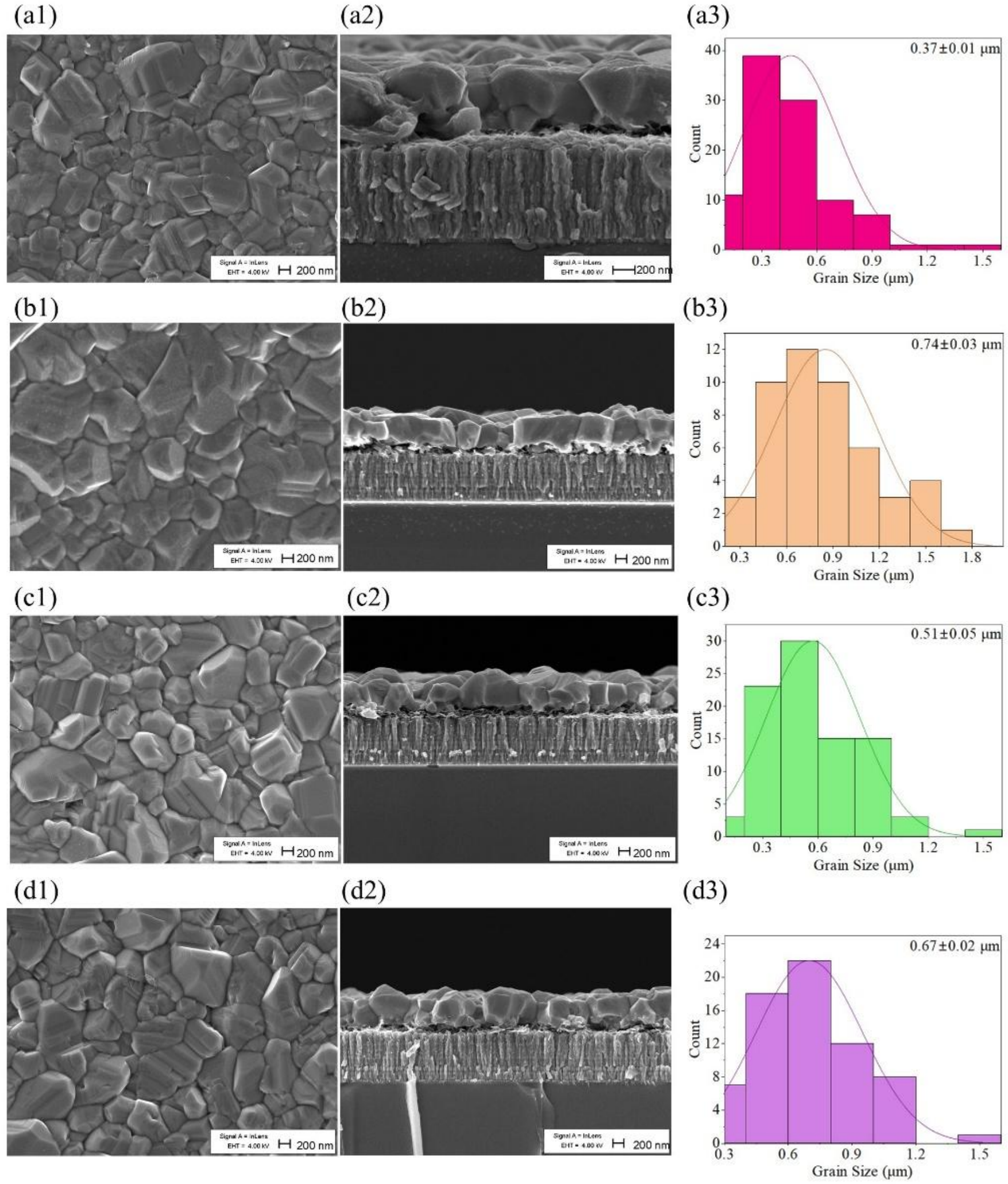

Figure S2. Top-view SEM images, cross-sectional SEM images, and corresponding grain size distributions for (a1–a3) Profile-1, (b1–b3) Profile-2, (c1–c3) Profile-3, and (d1–d3) Profile-4 sulfurization processes.

Although the 0.74 µm grains obtained under the Profile-2 condition represent the most favorable morphology in the present study, this grain size remains substantially smaller than the 2–3 µm grains commonly reported for CZTSSe absorbers. This comparison highlights the continued need for further optimization to improve the crystalline quality of solution-processed CZTS absorber layer.[2–4] Furthermore, the elemental compositions of CZTS films obtained under different sulfurization profiles were analyzed by energy-dispersive X-ray spectroscopy (EDX), with the results summarized in Table S1 (Supporting Information). Among the four conditions, the Profile-2 films exhibit a Cu-poor/Zn-rich composition that closely matches the targeted precursor ratios of Cu/(Zn+Sn) = 0.85 and Zn/Sn = 1.2 (see the Experimental Section), which are widely regarded as favorable for high-performance CZTS absorbers.

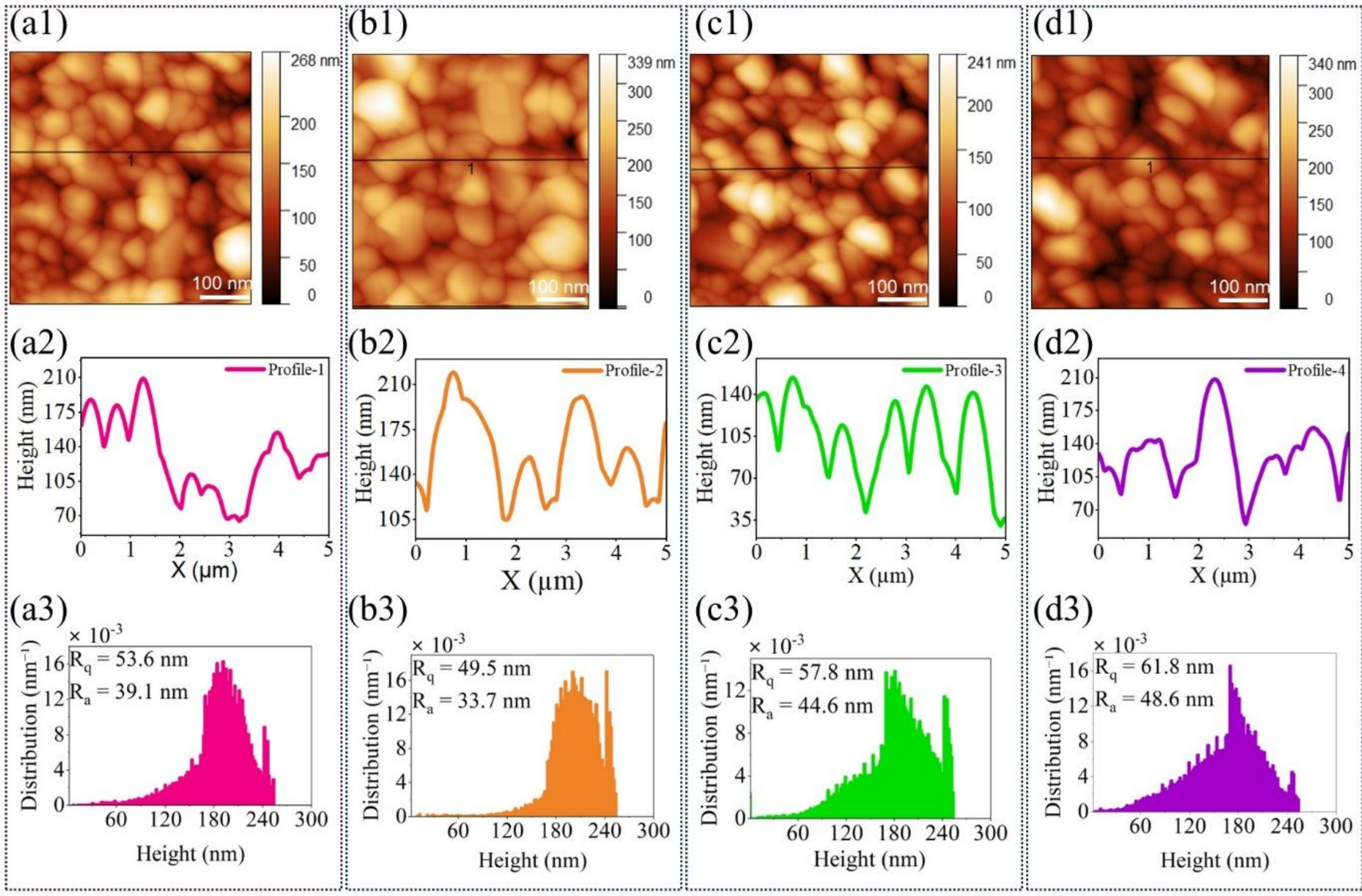

Figure S3. AFM analysis of CZTS absorber films prepared using different sulfurization profiles: (a1–d1) AFM topography images of Profile-1, Profile-2, Profile-3, and Profile-4, respectively; (a2–d2) corresponding surface-height line profiles; and (a3–d3) full-area surface-height distributions. The root-mean-square roughness (R_q) and arithmetic average roughness (R_a) were calculated over the entire AFM scanning area.

Table S1. Elemental composition of the pure CZTS thin film (TF) with different sulfurization profiles examined by EDX analysis.

CZTS	Composition, at%				Ratio	
Thin Film	Cu	Zn	Sn	S	Cu/(Zn+Sn)	Zn/Sn
Profile -1	18.6	11.6	9.1	60.5	0.89	1.28
Profile -2	18.9	11.7	9.8	59.4	0.87	1.18
Profile -3	19.1	12.1	9.1	59.5	0.90	1.32
Profile -4	19.5	12.5	9.3	59.5	0.89	1.34

To further investigate the surface morphology of CZTS thin films obtained under different sulfurization profiles, atomic force microscopy (AFM) measurements were performed. Figure S3a–d presents the corresponding AFM topography images and height profiles. The Profile-1 sample exhibits a surface height variation ranging from approximately 70 nm to 210 nm, comparable to that observed for the Profile-4 film. The Profile-3 sample shows reduced surface roughness, with a maximum height of ~140 nm. In contrast, the Profile-2 film displays the largest local surface height variation, exceeding 210 nm. To provide a quantitative comparison over the

entire AFM scanning area, the root-mean-square roughness (R_q) and arithmetic average roughness (R_a) were calculated. The R_q/R_a values are 53.6/39.1, 49.5/33.7, 57.8/44.6, and 61.8/48.6 nm for Profile-1, Profile-2, Profile-3, and Profile-4, respectively. Despite the larger local height variation observed along the selected line profile, Profile-2 exhibits the lowest full-area surface roughness, with R_q = 49.5 nm and R_a = 33.7 nm, indicating a comparatively smoother surface morphology. Furthermore, the Raman spectra were used to analyze the crystalline CZTS formation under the four sulfurization conditions as presented in Figure S4. In all cases, crystalline CZTS formation is confirmed by the presence of the dominant Raman peak at 338 cm^{-1}, corresponding to the A_1 vibrational mode of the kesterite CZTS structure, along with a weaker peak at 287 cm^{-1}. X-ray diffraction (XRD) analysis (Figure S5) further confirmed the structural characteristics, showing prominent diffraction peaks at 18.3°, 28.4°, 33.0°, 47.3°, and 56.2°, which correspond to the (101), (112), (200), (220), and (312) planes of the kesterite CZTS phase, consistent with literature reports.[1,2,6] From both Raman and XRD analysis, it is evidenced that the sulfurization profile does not significantly change the main peak of Raman at 338 cm^{-1} and the dominant XRD (112) reflection 28.4°. For un-sulfurized samples (Reference), the precursor film was also included in Figures S3 and S4. These spectra show no clear signatures of crystalline CZTS, confirming that the precursor layer was predominantly amorphous prior to sulfurization. Minor features attributable to ZnS and SnS secondary phases were detected in all sulfurized samples, indicating their presence across the different sulfurization conditions.

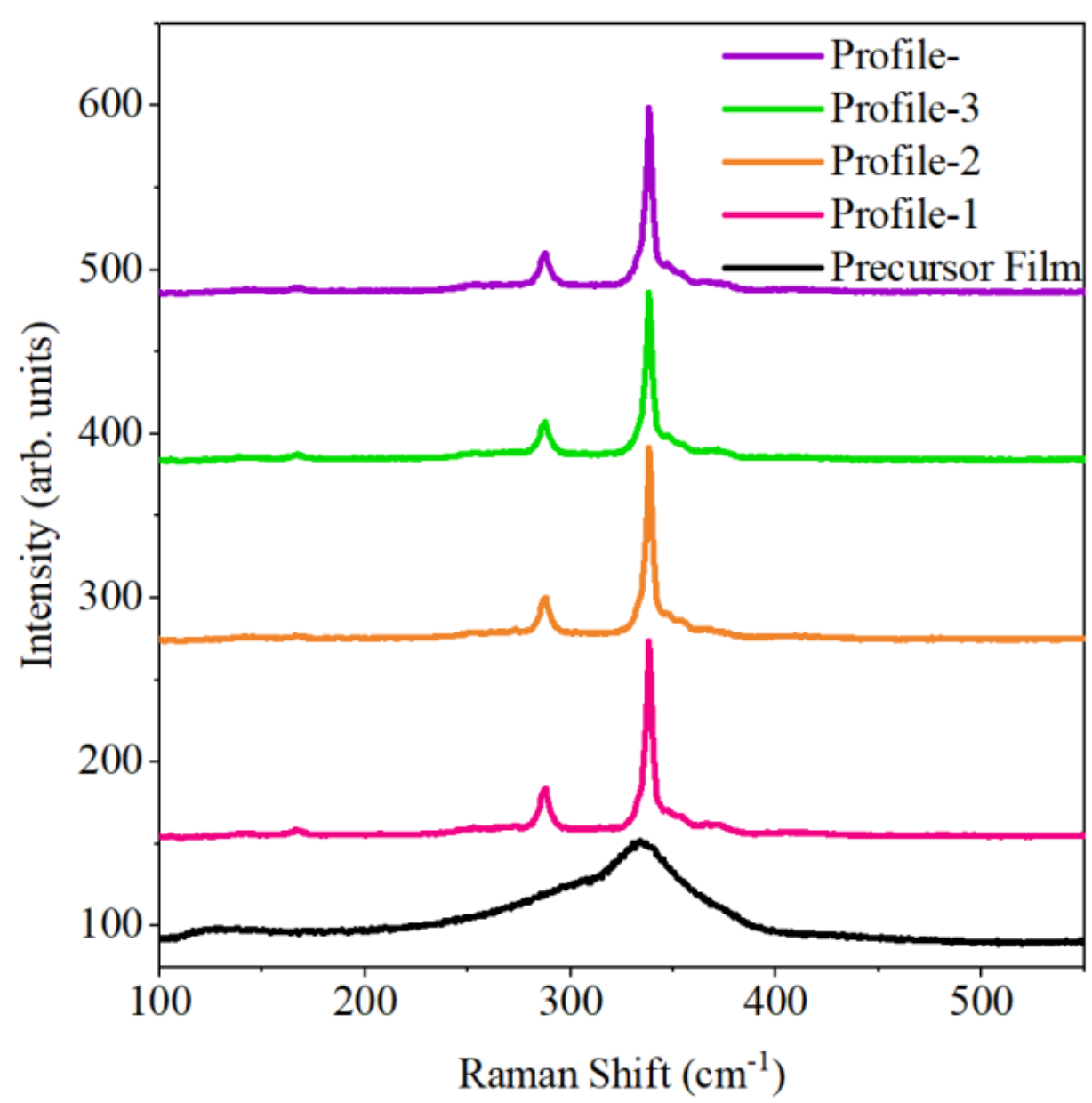

Figure S4. Raman result of CZTS precursor film and CZTS thin film with different sulfurization profiles.

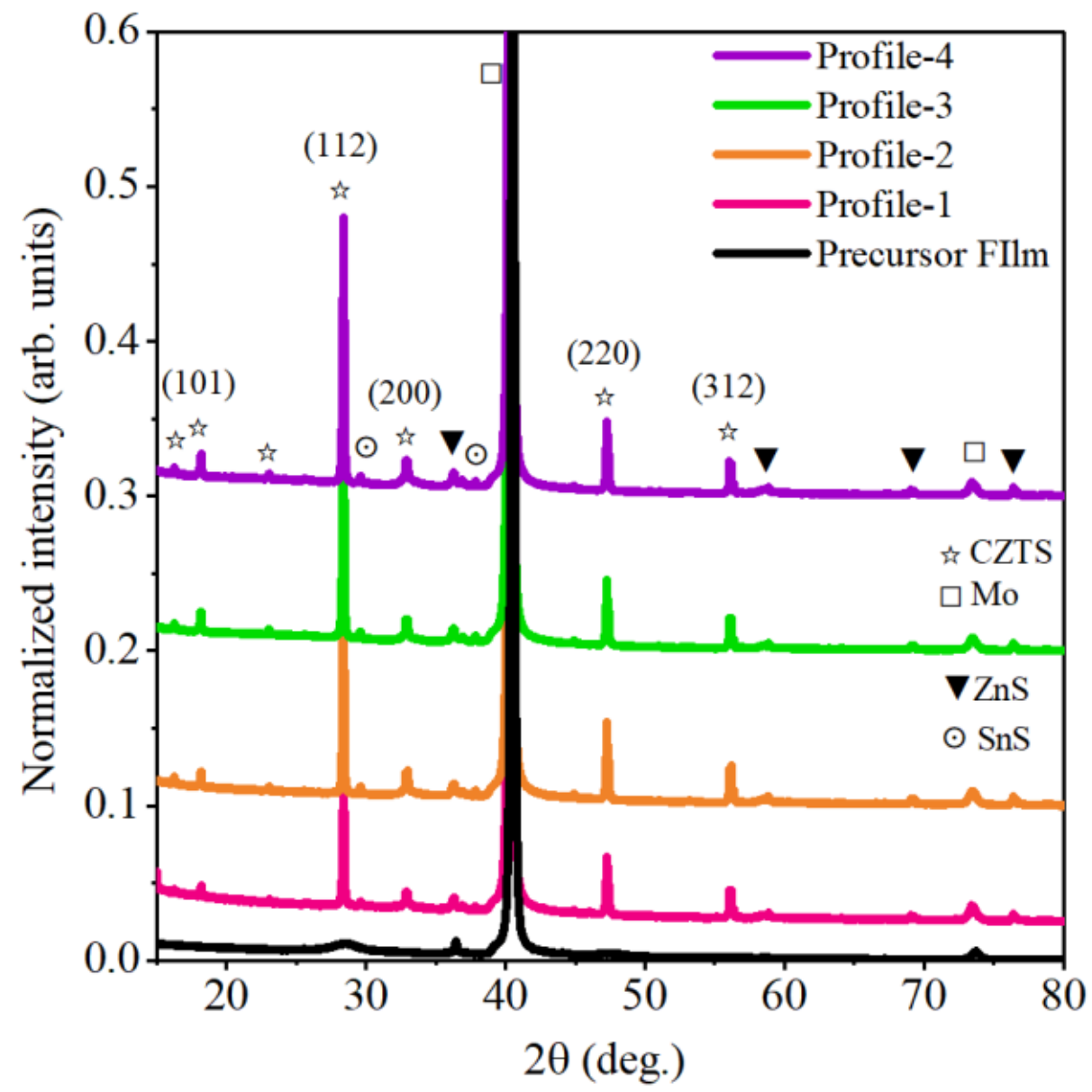

Figure S5. XRD result of CZTS precursor film and CZTS thin film with different sulfurization profiles.

To evaluate the electrical performance of CZTS solar cells associated with different sulfurization profiles, current–voltage (I–V) measurements were performed under standard AM 1.5G illumination. Figure S6 shows the statistical distribution of the key photovoltaic parameters (a short-circuit current density (J_{SC}), an open circuit voltage (V_{OC}), a fill factor (FF), and a power conversion efficiency (PCE) extracted from 16 individual cells per device. Among the four sulfurization profiles, Profile-2 sample exhibits the highest performance, with a J_{SC} of 20 mA cm^{-2}, a V_{OC} of 721 mV, FF of 50.5% and a PCE of 7.3%. The enhanced device performance observed for Profile-2 sample correlates well with the pronounced grain coalescence observed in the SEM analysis. Larger grains and reduced grain boundary density are expected to suppress bulk recombination and facilitate more efficient charge transport, leading to improved current collection and voltage output. Interestingly, the statistical distribution of FF reveals that Profile-1 sample yields the highest average FF of ~54%, slightly higher than the ~50% observed for Profile-2 sample. This difference may arise from variations in series resistance or back-contact quality, which can influence FF independently of bulk grain morphology. Table S2 summarizes the device efficiencies for each sulfurization profile, yielding PCE values of 7.3% (Profile-2), 6.2% (Profile-1), 5.7% (Profile-3), and 5.2% (Profile-4). Notably, the 7.3% PCE achieved for sample sulfurized under Profile-2 conditions—using a DMSO-based CZTS precursor processed entirely in ambient air and without additional post-annealing, or doping/alloying treatments—is comparable to the 7.6% PCE reported for state-of-the-art air-processed CZTS devices.[7] Profile-2 sulfurization was therefore selected as the baseline to study post-annealing cooling effects. Overall, these results demonstrate that careful control of the sulfurization temperature profile plays a critical role in enhancing CZTS crystallinity, reducing bulk recombination losses and, ultimately improving CZTS solar cell performance.

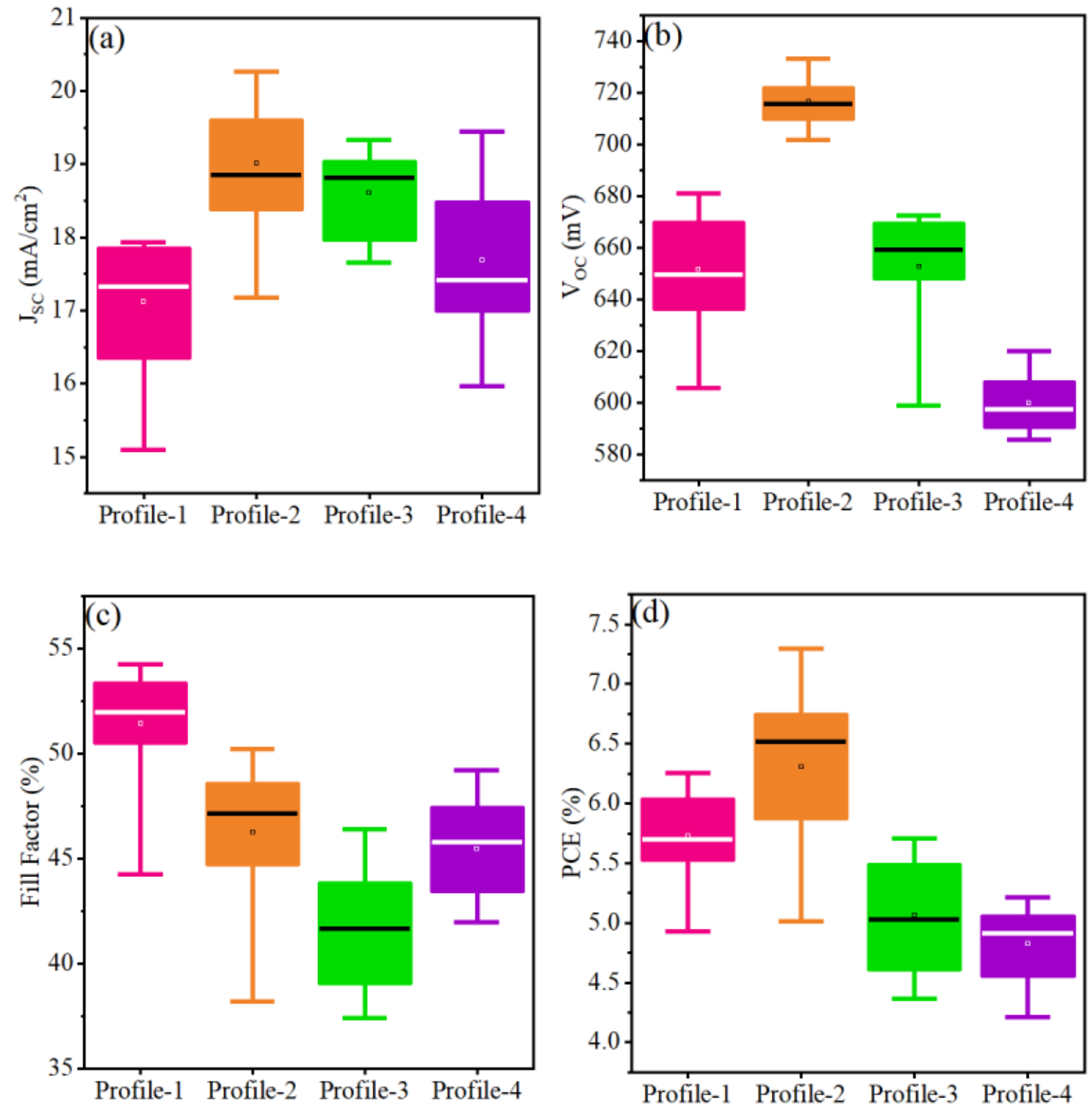

Figure S6. Box plot of the parameter of CZTS solar cells with different sulfurization profiles.

Table S2. Performance parameter of CZTS solar cells with different sulfurization profiles.

CZTS Device	J_{SC} (mA cm^{-2})	V_{OC} (mV)	FF (%)	PCE (%)
Profile-1	17.9	646	53.6	6.2
Profile-2	20.0	721	50.5	7.3
Profile-3	18.6	660	46.4	5.7
Profile-4	19.4	604	44.2	5.2

Supplementary Note S2: Single-diode model calculation to extract key solar cell parameters including the ideality factor (n), shunt resistance (R_{SH}), series resistance (R_S), and saturation current density (J_0) are summarized in Table 1, from the J–V characteristics.[8,9]

$$J = J_0 \left\{ \exp\left[\frac{q(V + JR_S)}{nkT}\right] - 1 \right\} + \frac{V + JR_S}{R_{sh}} - J_L \qquad (1)$$

Where J_0 is the saturation and J_L the photogenerated current densities. The thermal energy is expressed as kT, where T is the absolute temperature and k is the Boltzmann constant. The non-

ideal behaviour of the solar cell is explained by the diode ideality factor n, the series resistance R_s, and the parallel resistance R_{sh}.

Supplementary Note S3: Band gap calculation

The effective band gap energy (E_g^*) can be determined using the relation:[10]

$$EQE \approx K\alpha L_{eff} \approx A\left(E - E_g^*\right)^{1/2}/E \qquad (2)$$

Supplementary Note S4: Urbach Energy

The Urbach energy (E_U) was calculated to evaluate sub-band gap absorption, using EQE data for each device. The calculation, developed by Troviano et al.,[11] employs the following relation:

$$\ln[-\ln(1 - EQE)] = \ln a + \frac{hv}{E_U} \qquad (3)$$

Supplementary Note S5: Temperature-dependent J-V measurement

The activation energy was calculated from the temperature-dependent J-V measurements. The behaviour of the V_{OC} of the cells as a function of the temperature V_{OC} (T) can be described by equation[12]:

$$V_{OC} = \frac{E_A}{q} - \frac{nkT}{q}\ln\left(\frac{J_{00}}{J_L}\right) \qquad (4)$$

where E_A is the activation energy, q is the elementary charge, n is the ideality factor, k is the Boltzmann constant, T is the temperature, J_{00} is the reverse saturation current prefactor and J_L is the light generated photocurrent density. In the temperature range of 20-300 K, the V_{OC} versus T shows linear behaviour. The extrapolation of this linear part to T = 0 K indicates the activation energy E_A.

Supplementary Note S6: Capacitance Voltage Measurement

Capacitance-voltage measurement is used to estimate the charge density (N_{CV}) and depletion region width (W_d) following equation.[13]

$$N_{CV} = \frac{-2}{qA^2\varepsilon_0\varepsilon}\left(\frac{d\left(1/_{C^2}\right)}{dV}\right)^{-1} \quad (5)$$

$$W_d = \frac{\varepsilon_0\varepsilon A}{C_0} \quad (6)$$

where ε, ε0, A, and C are the dielectric constants for CZTS thin-film solar cells, the permittivity of free space, the device area, and the measured capacitance for each applied DC bias, respectively. $(d(1/C^2)/dV)^{-1}$ is the slope value.

Supplementary Note S7: Admittance spectral measurement

Admittance spectral measurements are utilized to estimate the defect level. The temperature dependence of the inflection frequency ω_0 is presented by following equation:[14]

$$\omega_0(T) = 2\, N_{c,v}\, v_{th}\;\; \sigma_{n,p} \exp(\frac{-E_a}{kT}) = 2\, \xi_0 T^2 \exp(\frac{-E_a}{kT}) \quad (7)$$

where $N_{c,v}$ is the effective density of states in the conduction and valence band, v_{th} is the thermal velocity, $\sigma_{n,p}$ represents the capture cross-section for electrons and holes. The emission pre-factor ξ_0 covers all the temperature independent parameters. E_a is the activation energy of the defect with respect to the corresponding band edge. The inflection frequency ω_0 corresponds to maximum of -ωdC/dω vs ω variation.

The systematic decrease in activation energy from 222 to 136 meV in this result therefore indicates that rapid cooling suppresses deeper or barrier-associated responses and shifts the device toward a shallower acceptor-dominated behavior, in agreement with reduced electronic losses and improved junction quality.

Supplementary Note S8: Conduction band offset (CBO)

Ultraviolet photoelectron spectroscopy (UPS) employed to determine the secondary-electron cutoff (E_{cutoff}) and valence-band onset (E_{onset}) for the ref and RCP devices. For CZTS, E_{cutoff} was 16.84 eV (Ref) and 16.17 eV (RCP), while for CdS it was 16.94 eV (Ref) and 16.51 eV (RCP). The corresponding E_{onset}values were 0.23 eV (CZTS, Ref) and 0.14 eV (CZTS, RCP), and 1.38 eV (CdS, Ref) and 1.39 eV (CdS, RCP). Using these parameters, the valence-band maximum (E_V) and conduction-band minimum (E_C) positions were subsequently derived according to the equations below.[15–17]

$$\Phi = hv - E_{cutoff} \quad (9)$$
$$E_V = E_{onset} + \Phi \quad (10)$$
$$E_C = E_V - E_g \quad (11)$$

Band alignment at the CdS/ CZTS interface is typically classified as either cliff-type or spike-type. In the cliff-type configuration, the buffer layer has a higher electron affinity than the absorber, resulting in a negative conduction band offset (CBO). Such a band alignment enhances interfacial recombination by facilitating electron accumulation at the junction, thereby reducing the V_{OC} and *FF*. Conversely, when the buffer layer has a lower electron affinity than the absorber, a positive CBO is formed, leading to a spike-type band alignment. A small spike can suppress interfacial recombination by limiting electron backflow while still allowing efficient carrier transport.

Supplementary Note S8: Temperature-dependent Photoluminescence

Asymmetric double sigmoidal (ADS) [18]

$$I(E) = A \cdot \left[1/\left(1 + \exp\left(-\frac{E-E_1+(W_1/2)}{W_2}\right)\right)\right] \cdot \left[1 - 1/\left(1 + \exp\left(-\frac{E-E_1+(W_1/2)}{W_3}\right)\right)\right] \quad (12)$$

Here, A, E_1, E_2, W_1, W_2 and W_3 are fitting parameters, where A corresponds to the intensity, E_1 to the peak position, and W_1 to the overall band width. Parameters W_2 and W_3 describe the low- and high-energy sides of the PL band, respectively.

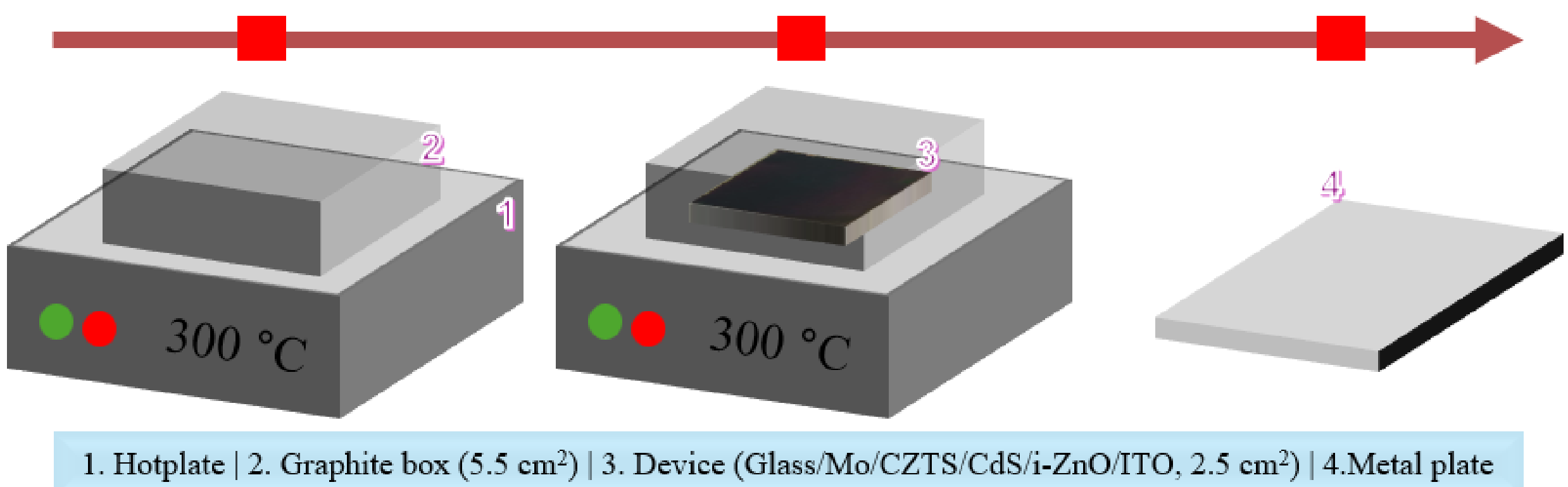

Figure S7. Schematic illustration of the device annealing procedures: (i) the hot plate and graphite box are preheated together; (ii) once the temperature reaches 300 °C, the device is placed inside the graphite box and the lid is closed to maintain a stable temperature; (iii) after annealing for 12 min, the device is removed and placed on a metal plate for cooling.

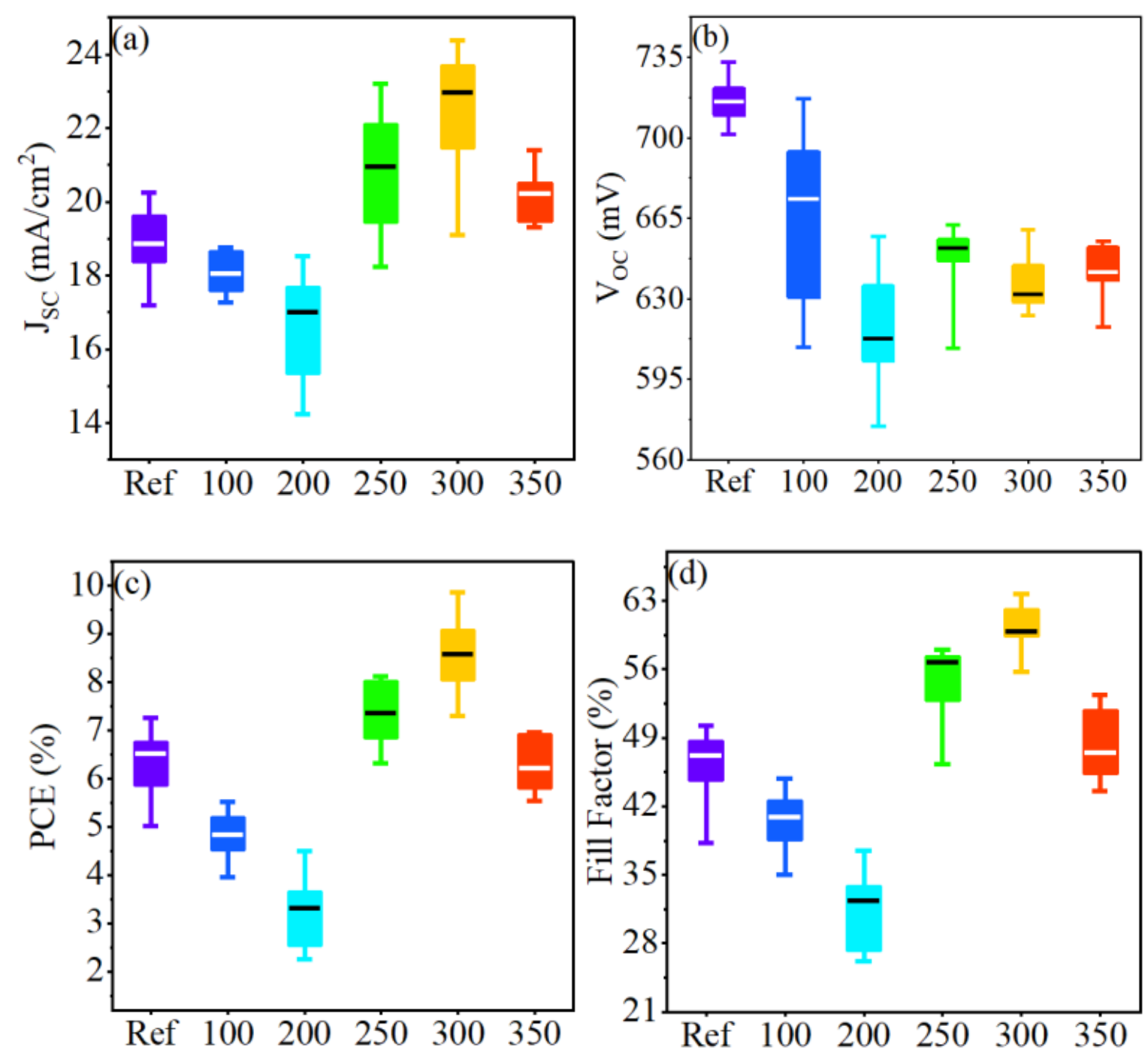

Figure S8. Box plot of the performance parameter of CZTS solar cells with different temperature of device annealing.

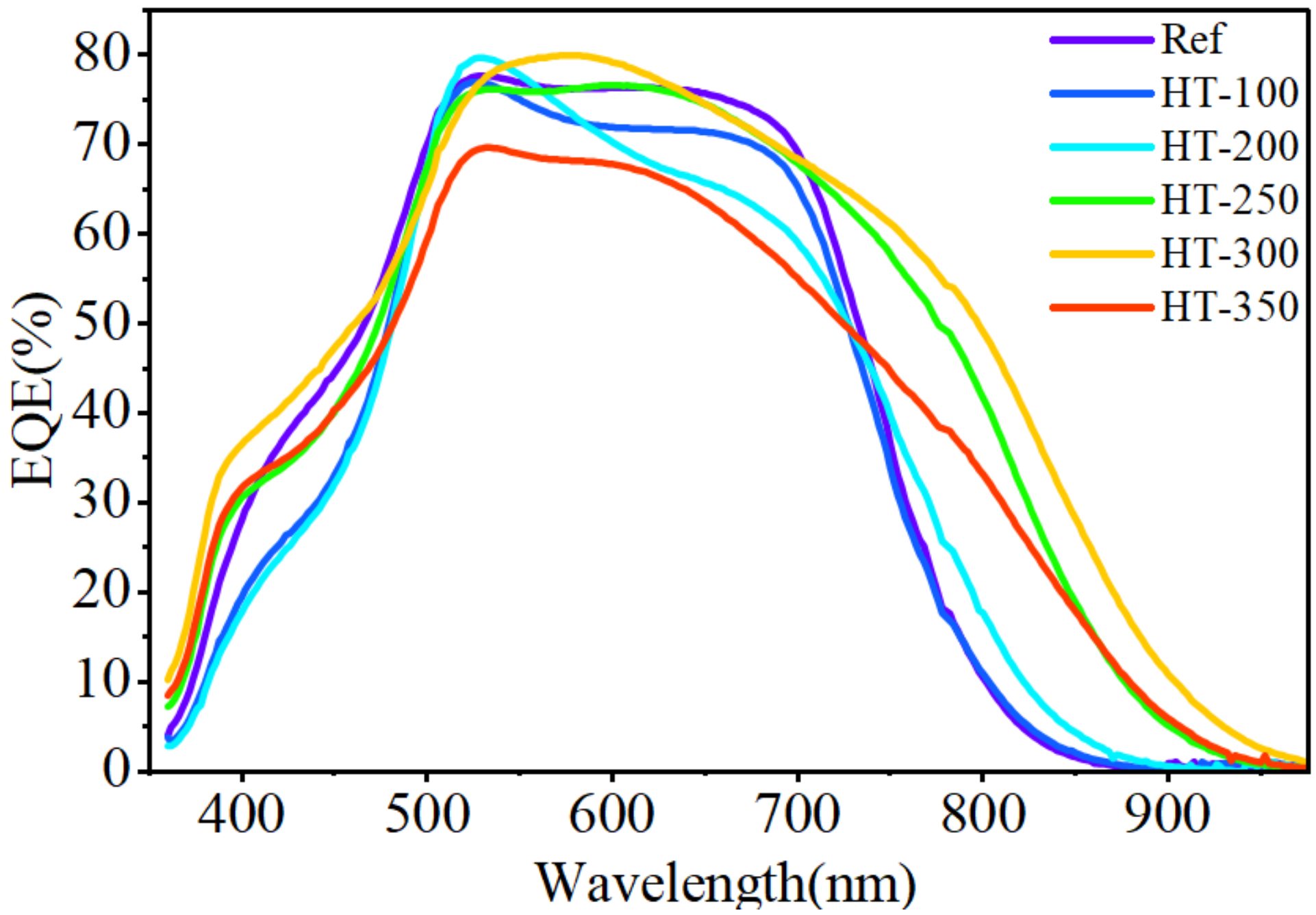

Figure S9. EQE result of CZTS solar cells with different temperature of device annealing.

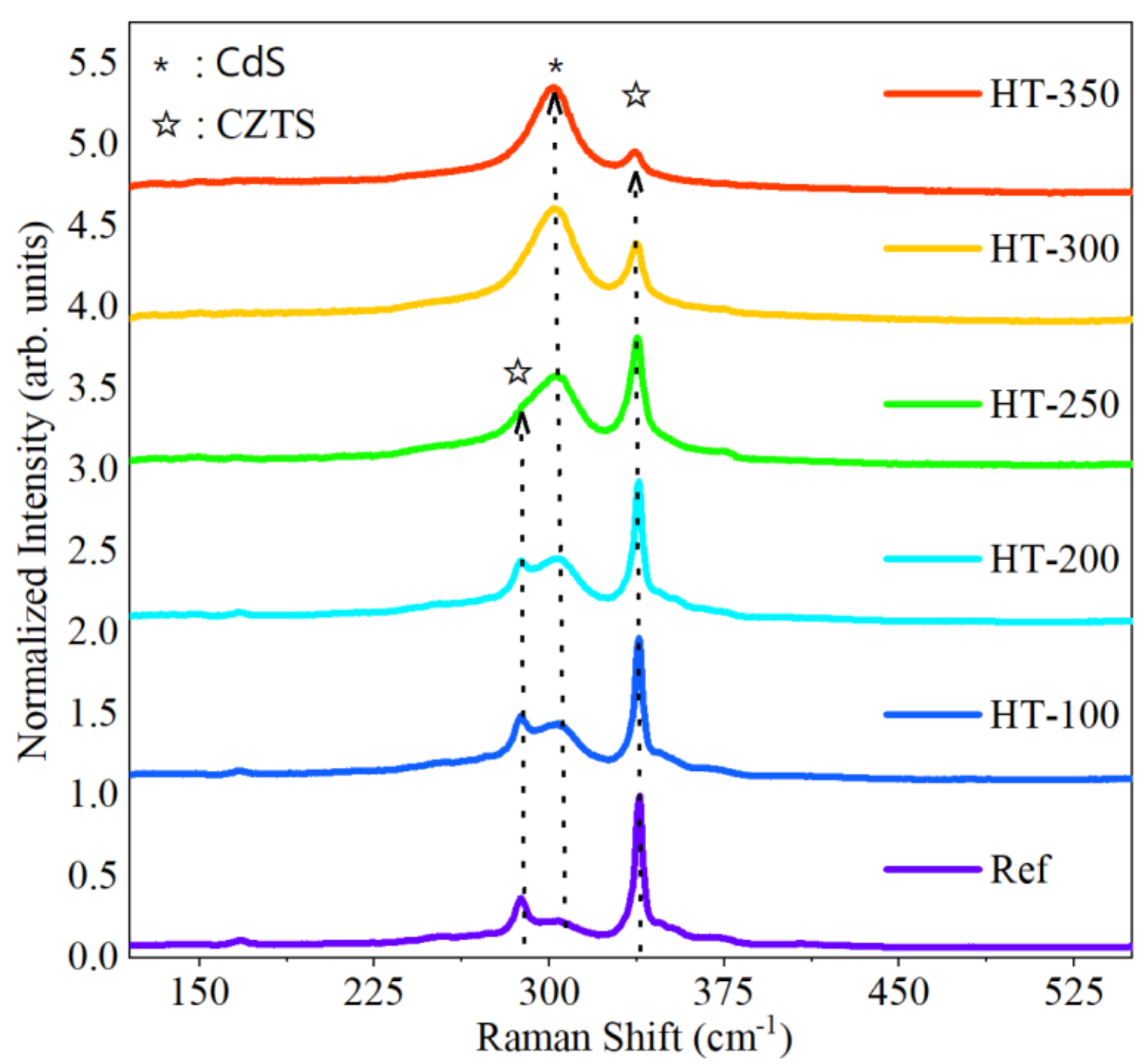

Figure S10. Raman result of the CZTS solar cells with different temperature of device annealing.

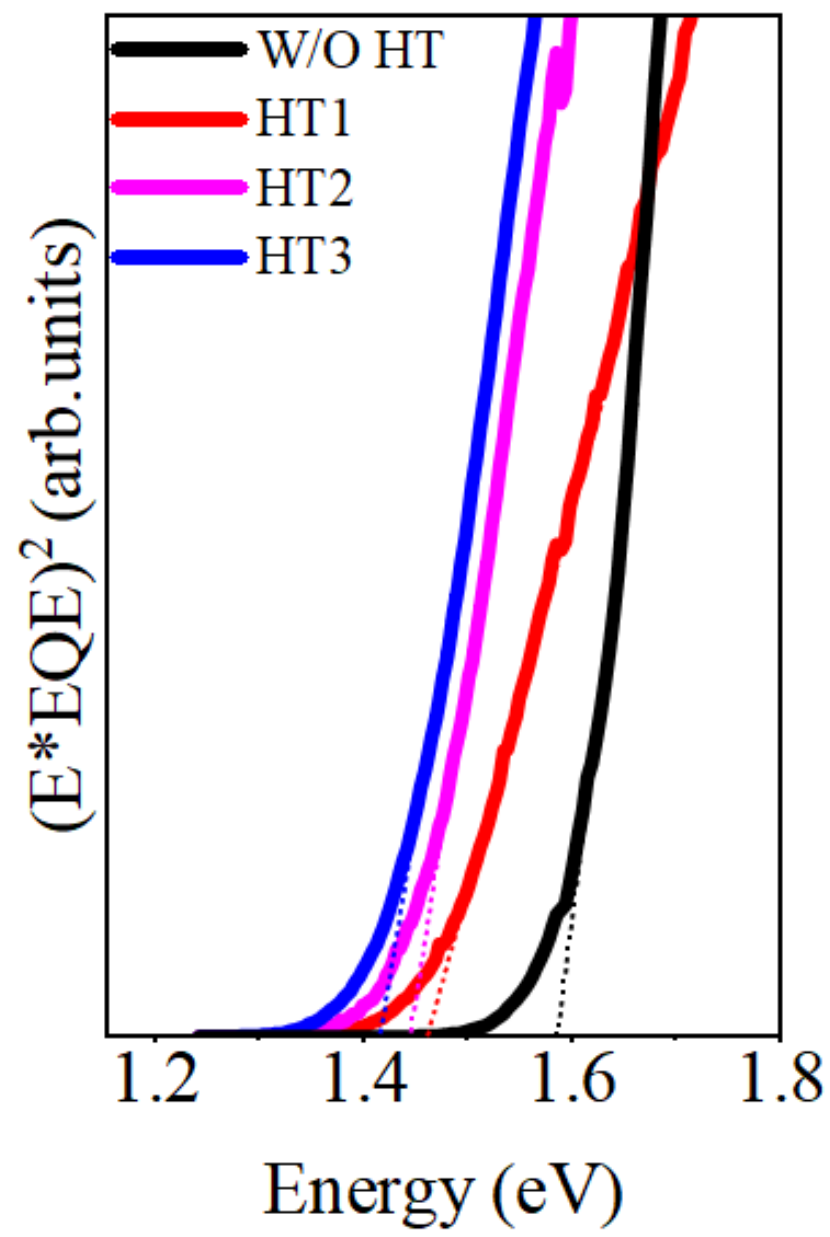

Figure S11. Bandgaps of CZTS devices extracted from EQE using the $(E \cdot EQE)^2$method. The bandgaps for Ref, HT1, HT2, and HT3 are 1.586, 1.462, 1.447, and 1.435 eV, respectively. Ref denotes the device without annealing; HT1 corresponds to annealing performed after sulfurization; HT2 corresponds to annealing performed after CdS buffer deposition; and HT3 corresponds to annealing performed after completion of the full device stack. All annealing treatments were carried out at 300 °C for 12 min followed by rapid cooling in ambient air.

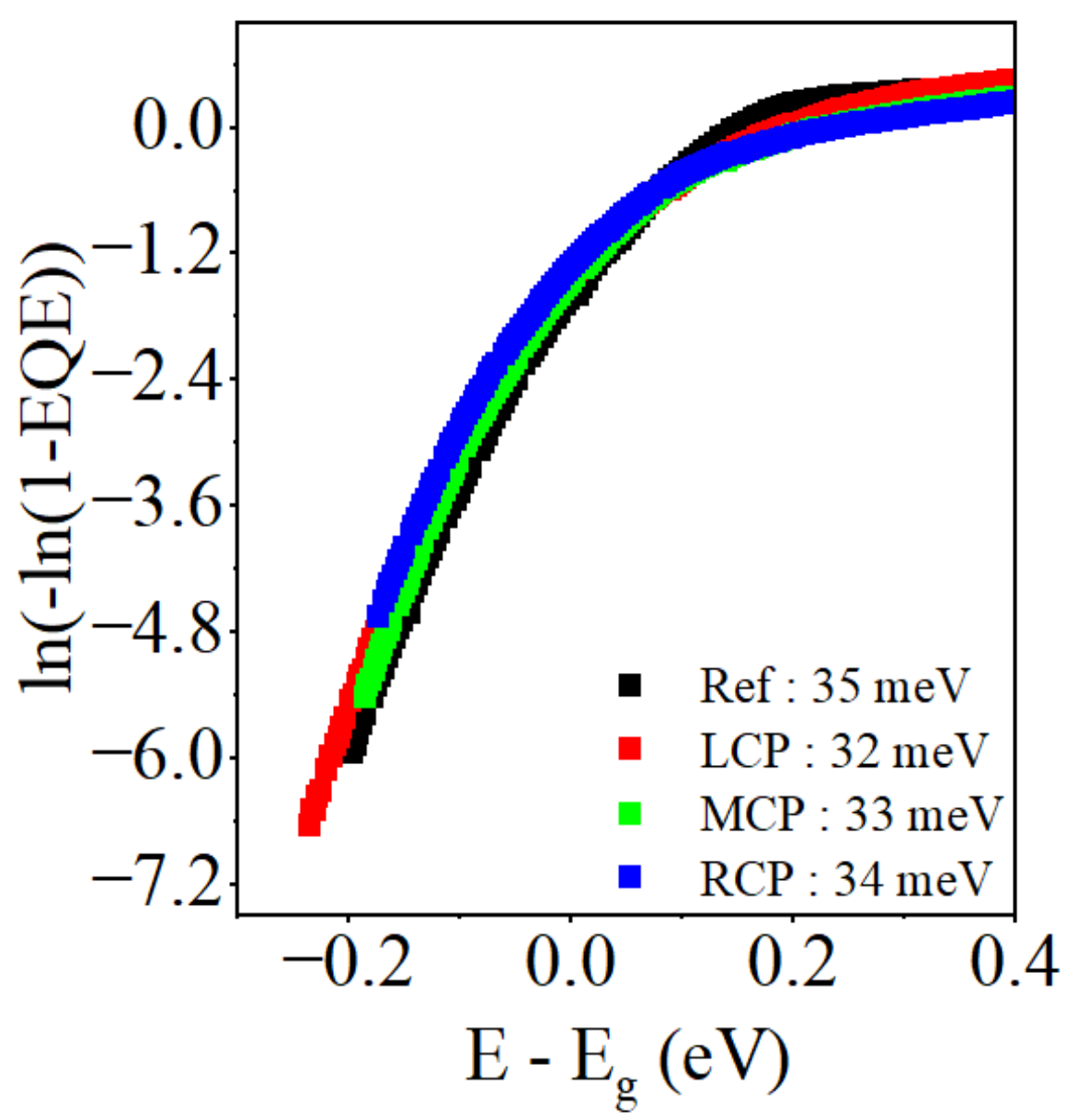

Figure S12. Urbach Energy (E_U) extracted from ln(-ln(1-EQE)) versus E-E_g plots

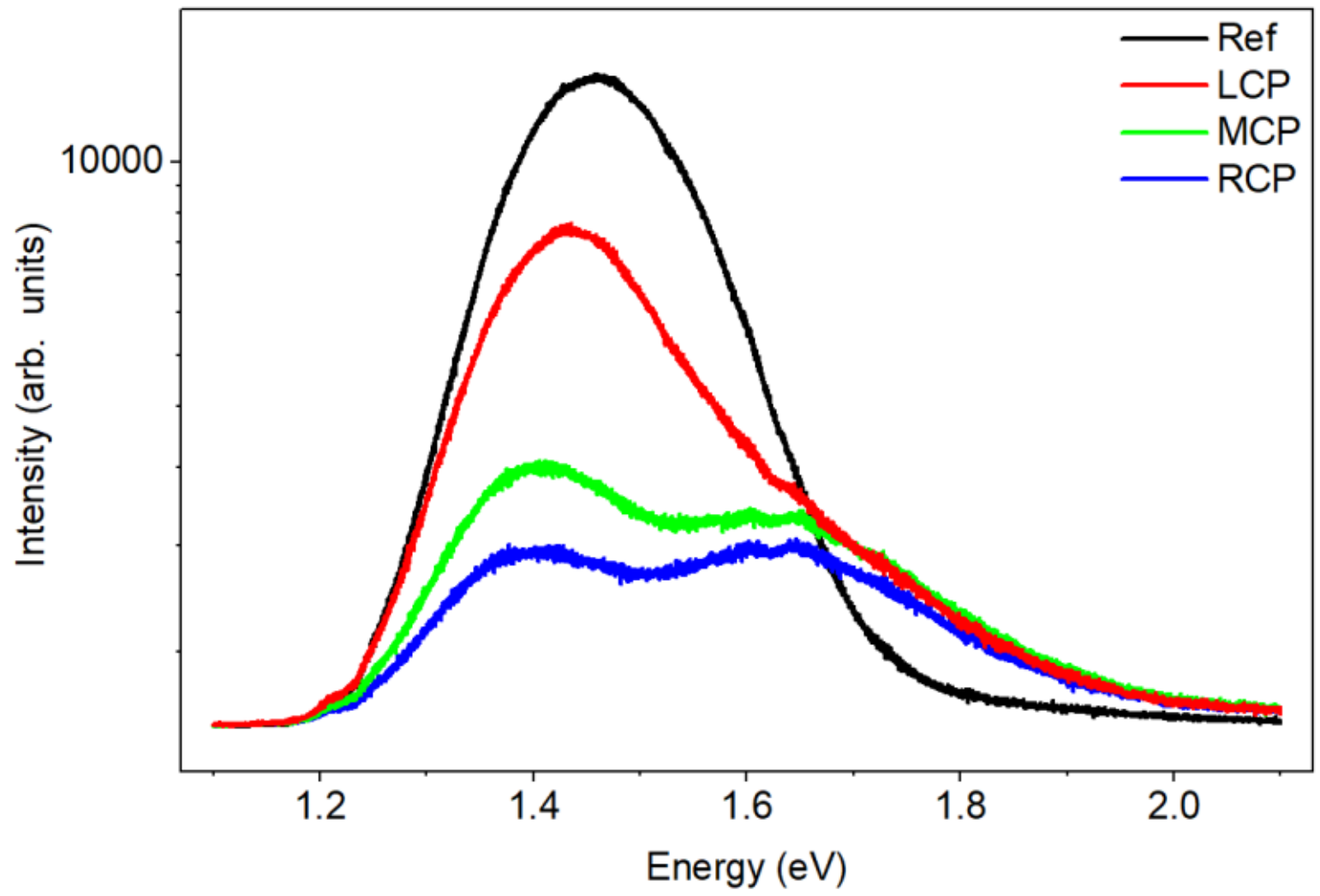

Figure S13. Photoluminescence (PL) spectra of the reference, LCP, MCP, and RCP devices in non-normalized scale.

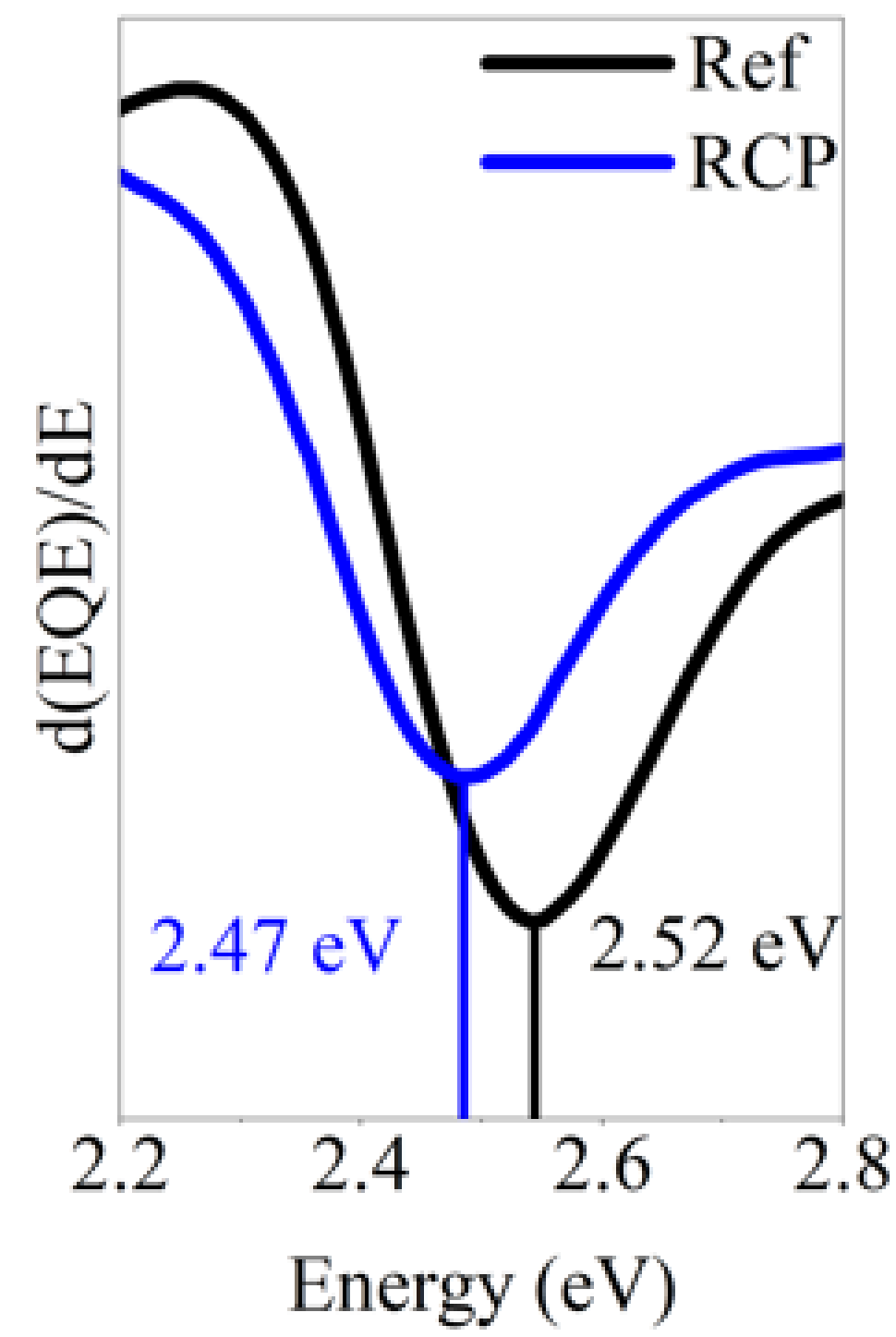

Figure S14. Bandgap of CdS for Ref and RCP devices calculated by d(EQE)/dE.

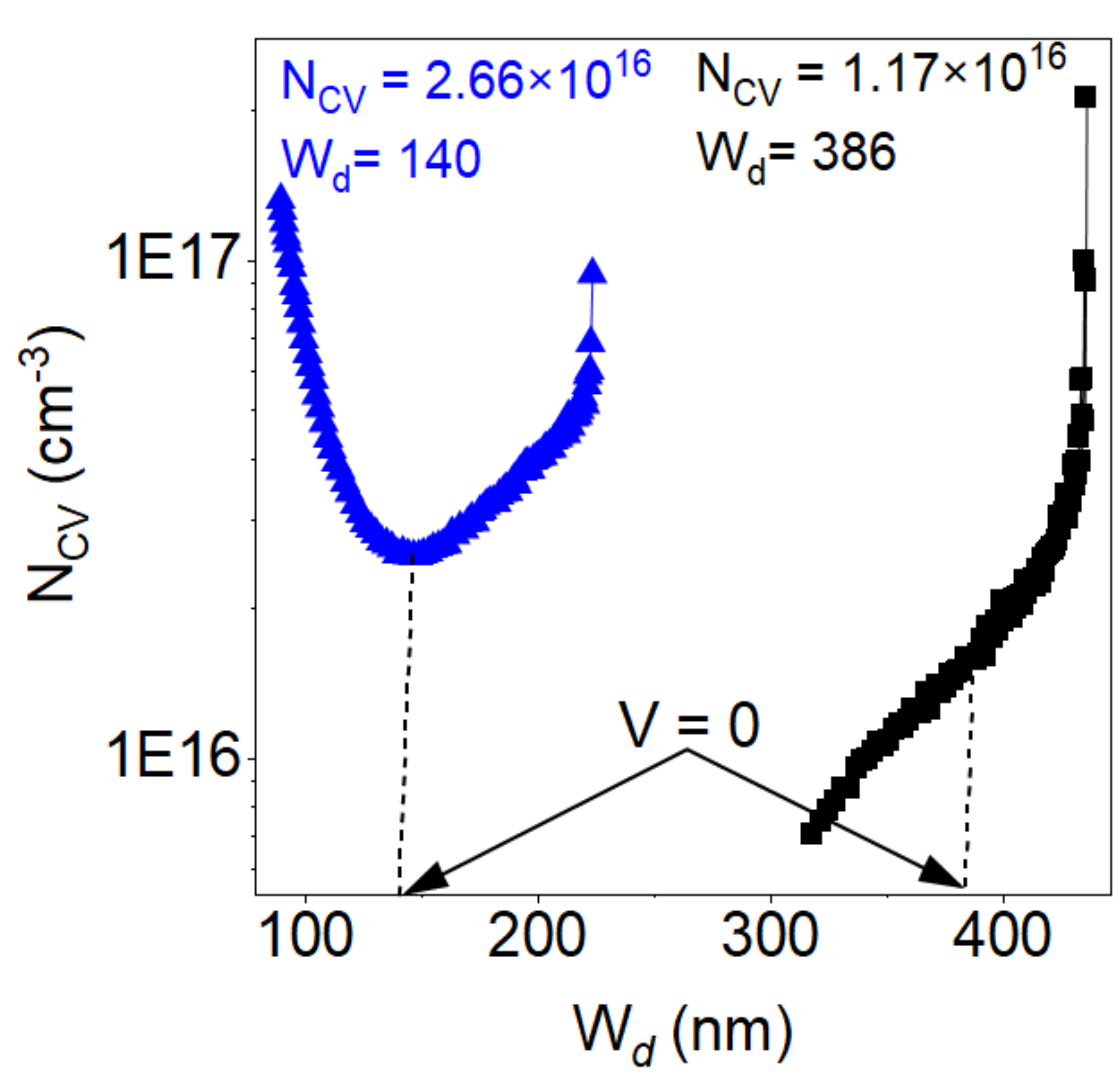

Figure S15. CV. Charge profiles of the Ref, and RCP devices measured by C–V measurement (NCV) (at 100 kHz).

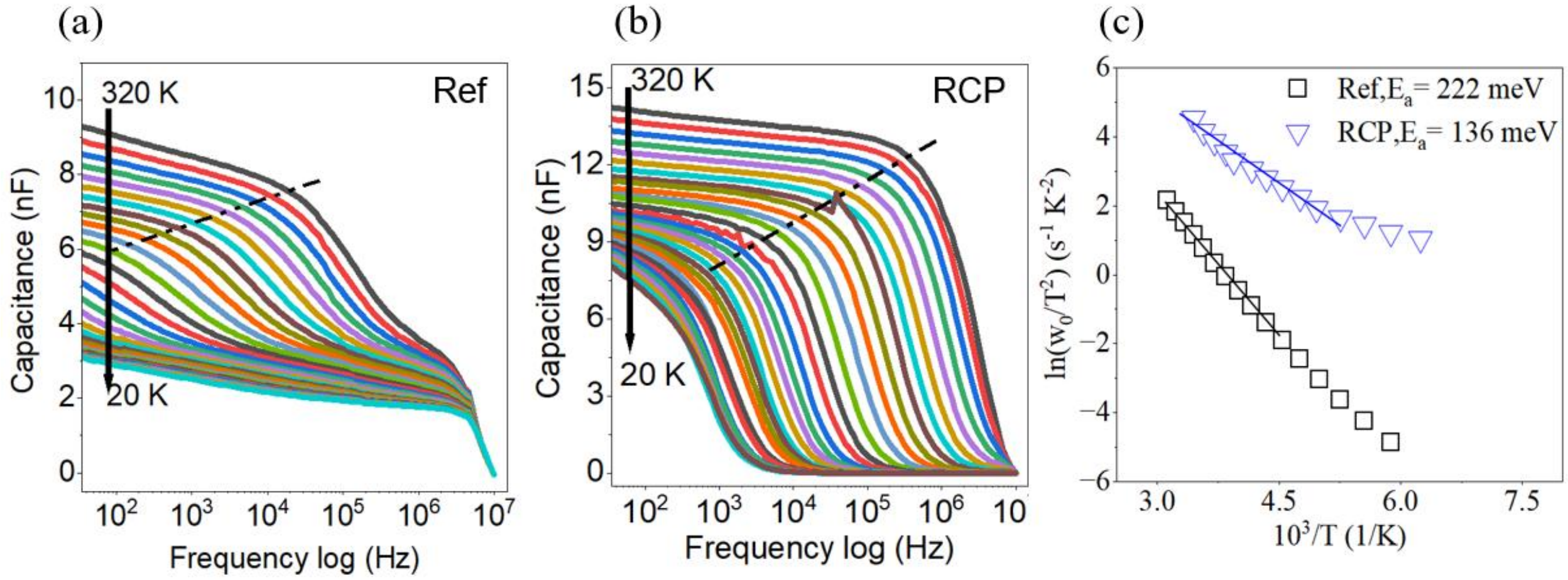

Figure S16. Admittance spectra of ref and RCP CZTS devices in the temperature range of 20–300 K, which probes frequencies from 20Hz to 2 MHz. (a) ref device and (b) RCP device. (c) Arrhenius plots used to extract the defect activation energy (E_a) for the Ref and RCP devices.

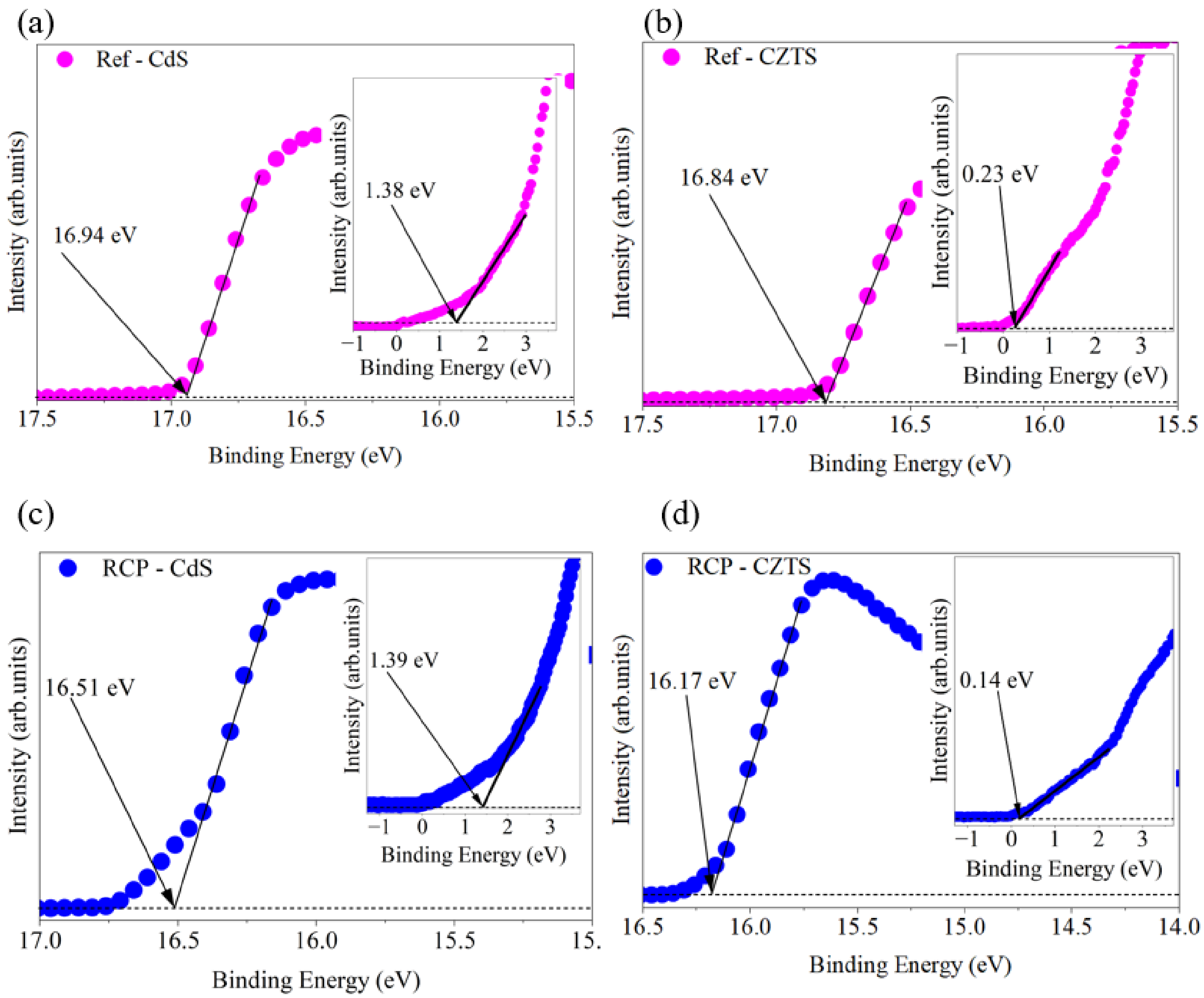

Figure S17. UPS spectra of (a) CdS in the Ref device, (b) CZTS in the Ref device, (c) CdS in the RCP device and (d) CTZS in the RCP device. The black solid line is the linear fitting, and black dashed line is for visual guidance.

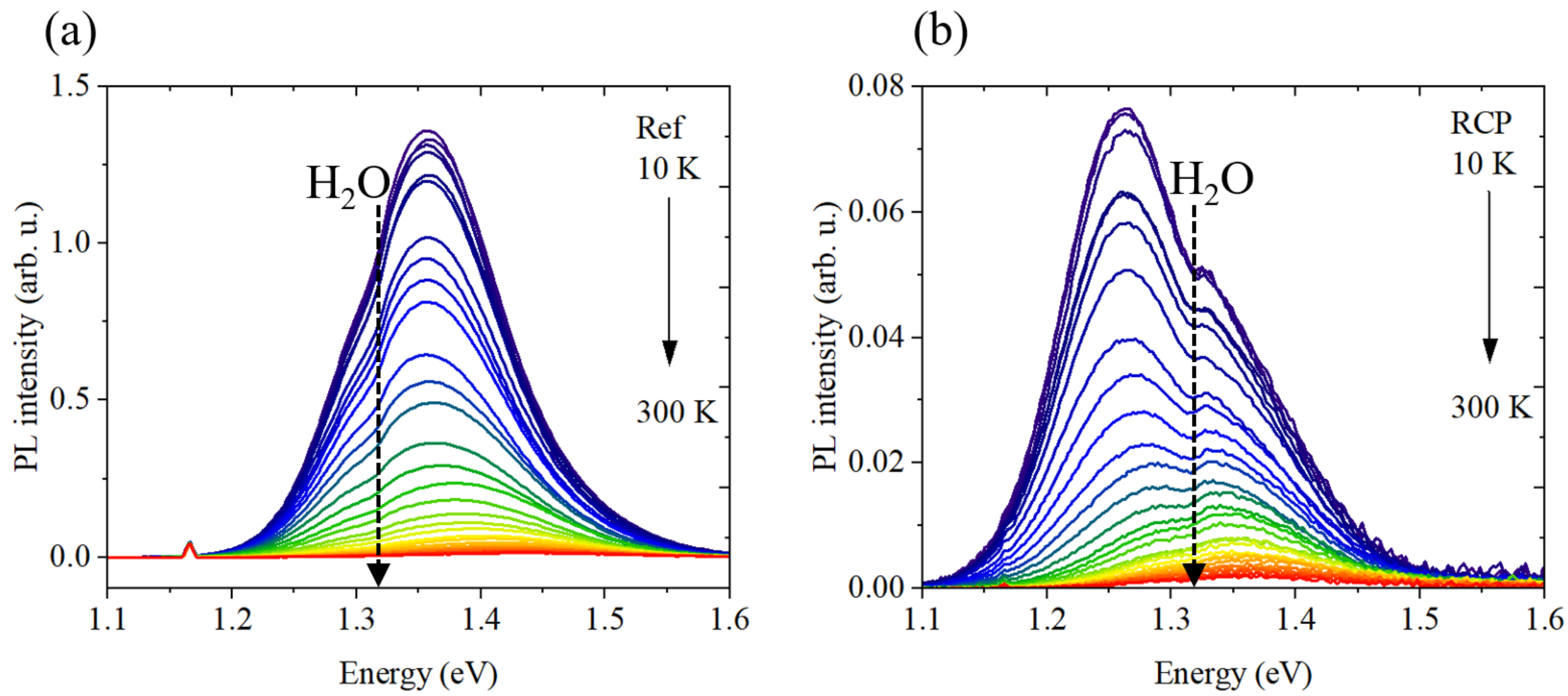

Figure S18. Temperature-dependent Photoluminescence measurement for Ref and RCP devices.

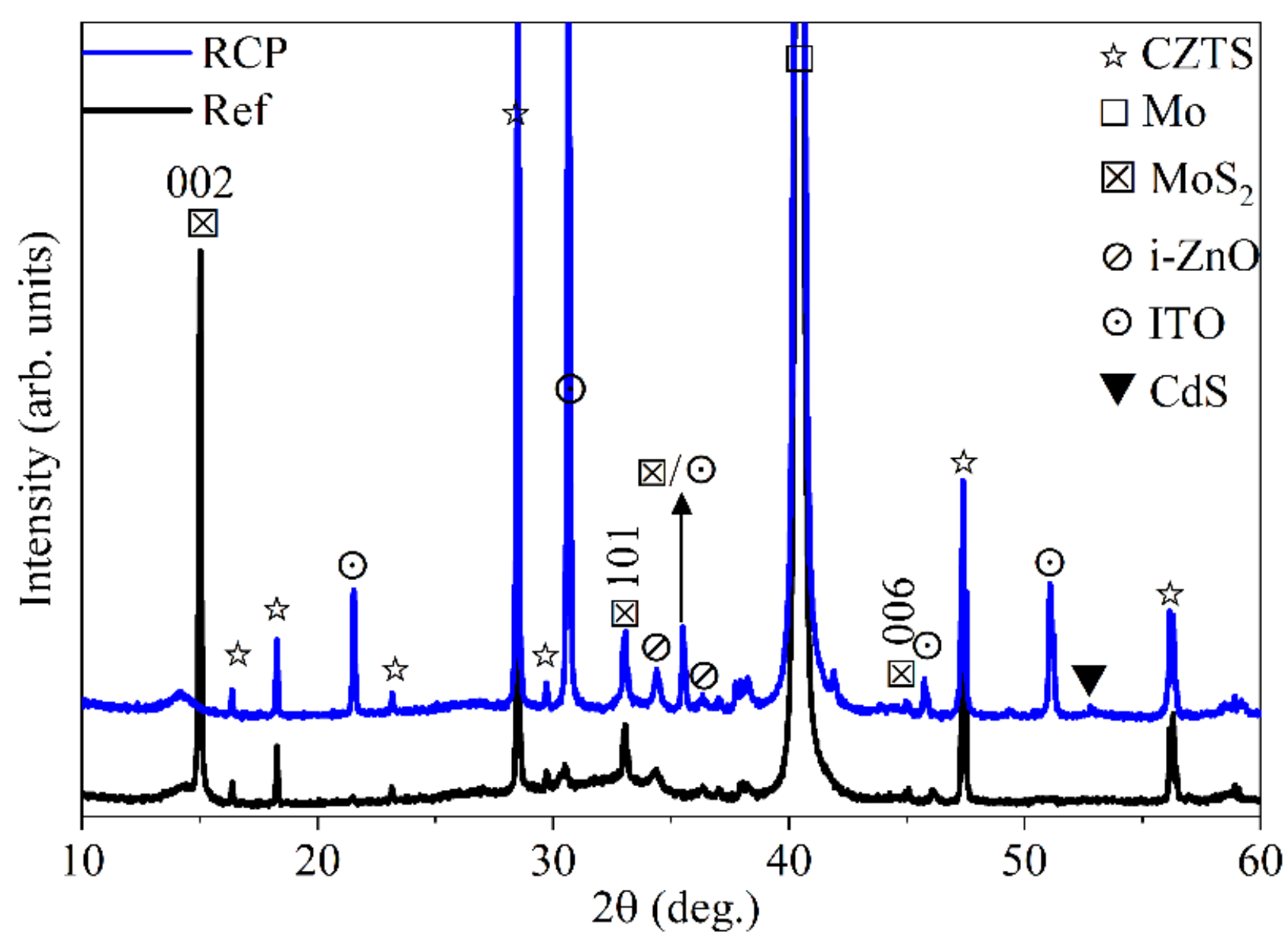

Figure S19. XRD result of the ref and RCP devices.

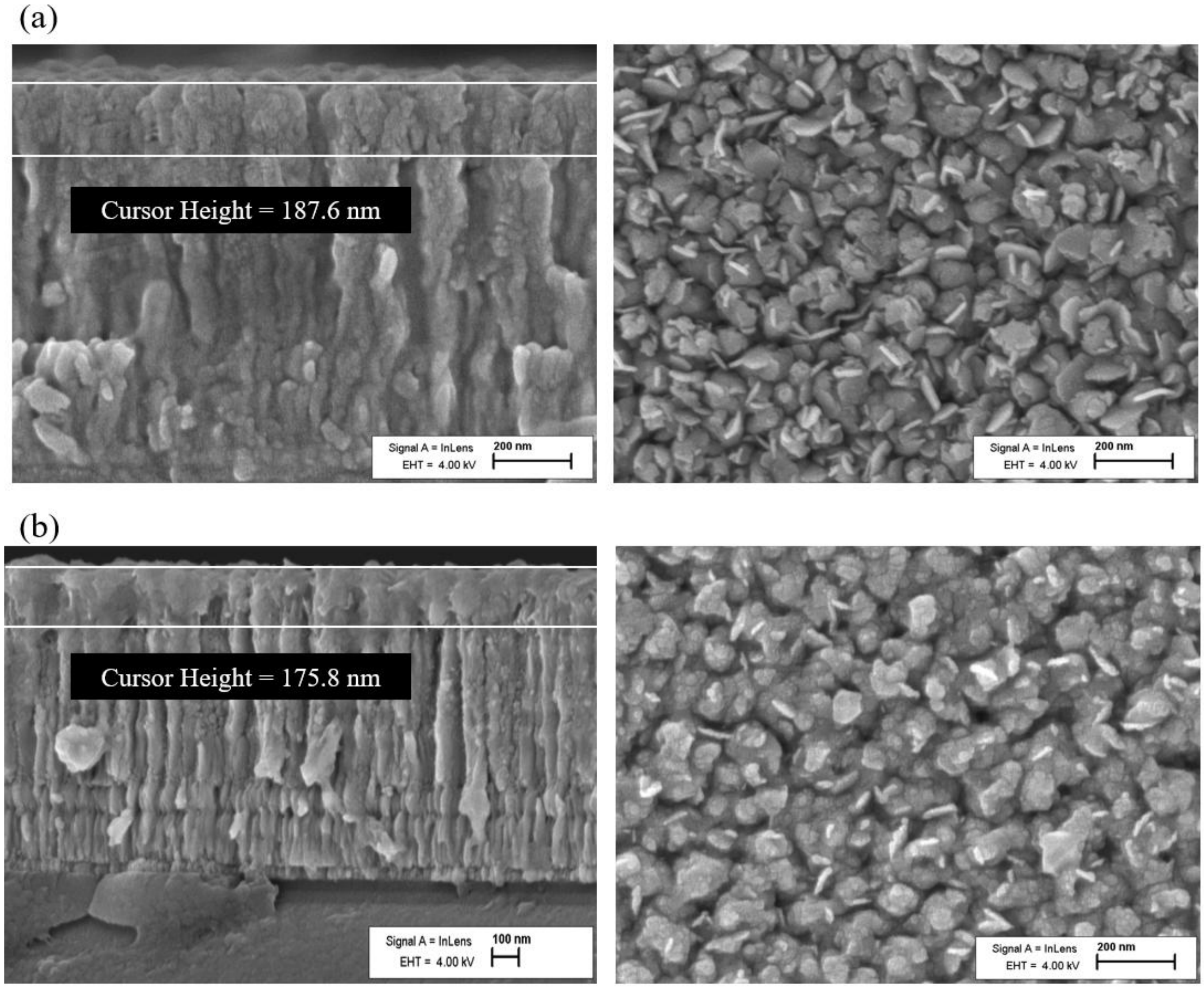

Figure S20. Cross-sectional SEM images and Top-view SEM images of soda-lime-glass/Mo after sulfurization. (a). Without annealing. (b) With annealing + RCP.

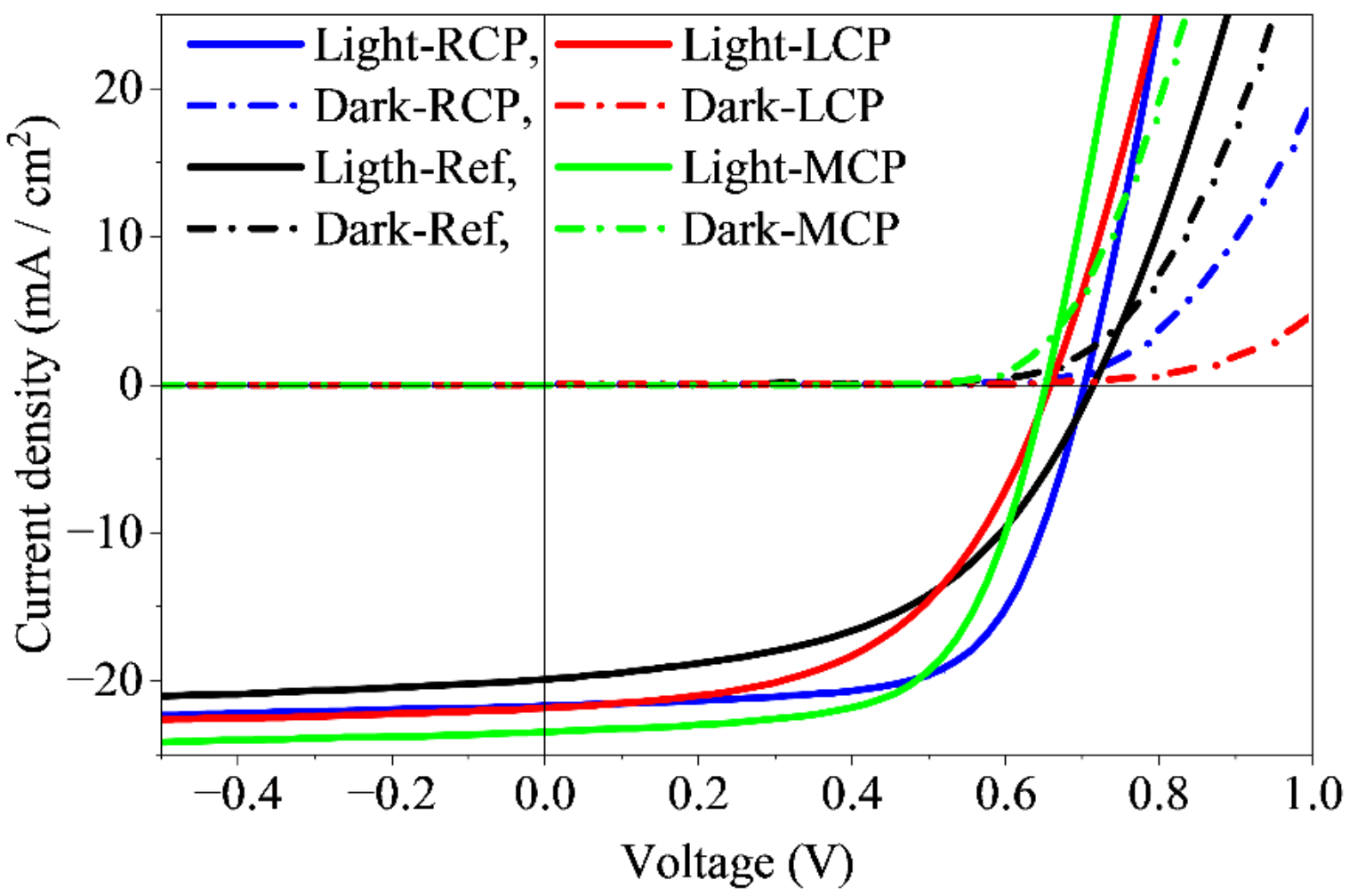

Figure S21. Light and dark J–V curves of the Ref, LCP, MCP, and RCP CZTS thin-film solar cells.

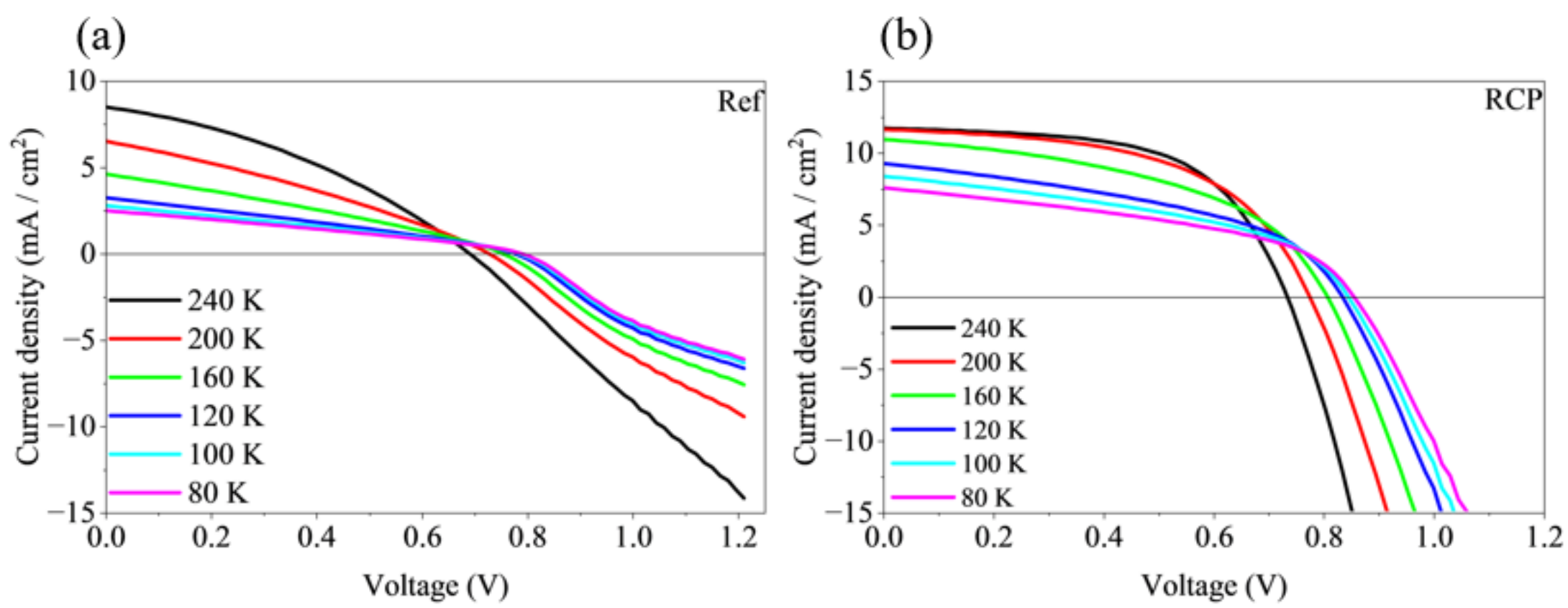

Figure S22. Temperature-dependent J–V curves of the (a) Ref and (b) RCP devices measured from 240 to 80 K.

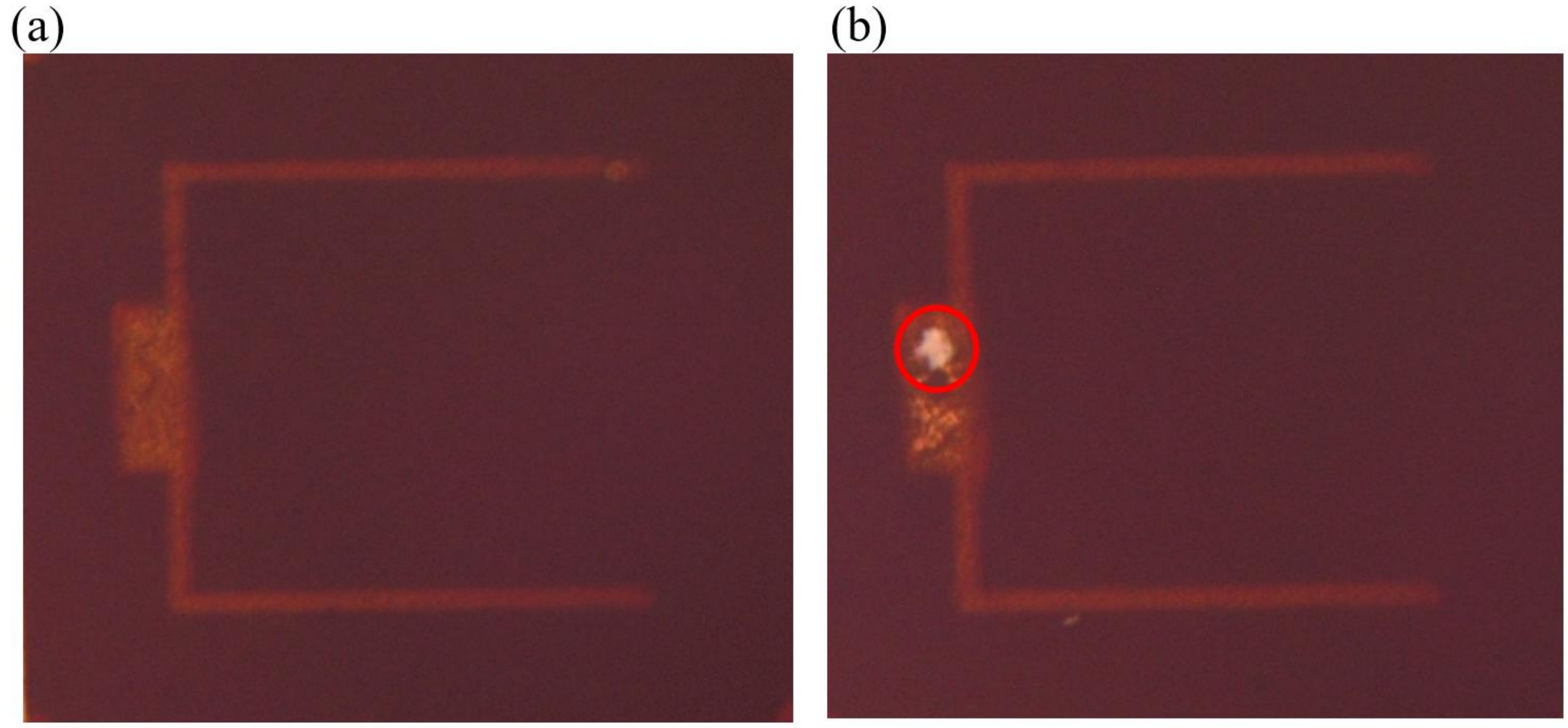

Figure S23. Au contact for the highest cell of RCP device (a) fresh and (b) repeated handling

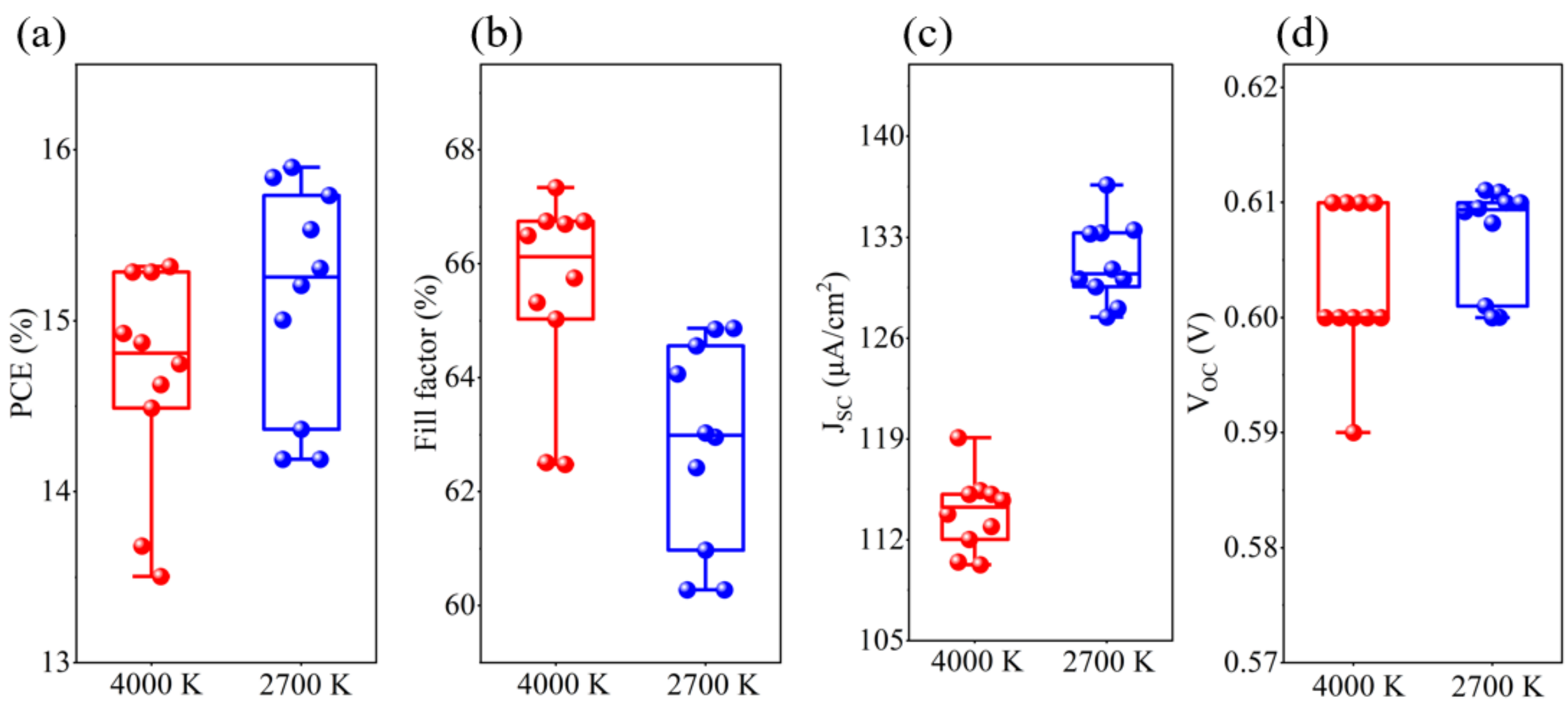

Figure S24. The statistics of (a) PCE, (b) FF, (c) J_{SC}, and (d) Voc of RCP device

Table S3. Performance parameter of CZTS solar cells with different temperature of device annealing.

Device Annealing Temperatures	J_{SC} (mA cm^{-2})	V_{OC} (mV)	FF (%)	PCE (%)
Ref	20.0	721	50.5	7.3

100 °C	18.2	679	44.8	5.5
200 °C	18.5	647	37.5	4.4
250 °C	21.7	653	57.1	8.1
300 °C	23.7	658	63.3	9.8
350 °C	20.5	654	51.9	7.0

Table S4. Photovoltaic parameters of the Ref, LCP, MCP, RCP CZTS thin-film solar cells at dark J-V measurement.

CZTS Solar Cells	n	J_0 [A/cm^2]	R_{sh} [Ω.cm^2]	R_s [Ω.cm^2]
Ref-Dark	2.7	1.3×10^{-7}	4.0×10^{5}	3.5
LCP-Dark	2.8	9.8×10^{-9}	7.0×10^{4}	8.8
MCP-Dark	2.2	4.6×10^{-8}	4.1×10^{4}	3.5
RCP-Dark	2.3	1.8×10^{-8}	7.3×10^{4}	5.6

Table S5. Indoor Performance of RCP device under varying illuminance levels under 4000 K and 2700 K LED illumination.

Samples	Illumination condition	Lux	PCE %	FF %	J_{SC} μA/cm^2	V_{OC} mV	Pin μW/cm^2	Pout μW/cm^2
RCP Device	4000 K	**1000**	**15.2**	**66.6**	**115.1**	**609.9**	**306.7**	**46.6**
		500	14.1	65.4	56.6	579.7	152.5	21.5
		200	11.7	58.5	22.2	549.8	60.97	7.1
	2700 K	**1000**	**15.9**	**64.8**	**133.2**	**609.9**	**331.6**	**52.7**
		500	15.5	63.4	68.4	593.7	165.8	25.7
		200	13.9	58.9	27.9	563.2	66.3	9.2

Table S6. Reproducibility of the 2700 K LED light source measured at approximately 1000 lux, showing the corresponding illuminance and power density over repeated measurements

Lux Value	Power density (mW/m^2)
1005	3253
1005	3255
1005	3269
1004	3264
1005	3262
1005	3259
1005	3273
1004	3268
1004	3264
1004	3263
1004	3274
1003	3263
1003	3263
1003	3273
1004	3269
1003	3272
1002	3265

References

1. Nasyori A, Pilvet M, Saar A, et al. In ambient air processed Cu2ZnSnS4 absorber layers from DMSO-based precursors: enhanced efficiency via device post-annealing. *J Mater Chem A*. 2025;13:30167-30179. doi:10.1039/D5TA04554A

2. Gong Y, Zhang Y, Zhu Q, et al. Identifying the origin of the: V ocdeficit of kesterite solar cells from the two grain growth mechanisms induced by Sn2+and Sn4+precursors in DMSO solution. *Energy Environ Sci*. 2021;14(4):2369-2380. doi:10.1039/d0ee03702h

3. Jian Y, Xie T, Han L, et al. Grain Growth Mechanism of CZTSSe Films Controlled by the Evaporation Area of Se. *Sol RRL*. 2023;7(9):1-12. doi:10.1002/solr.202300006

4. Jia S, Zhu W, Guo C, et al. Boron-Modified Heterointerface Boosts Kesterite Solar Cells to 0.58 V Open-Circuit Voltage. *Small*. 2025;21(42):1-14. doi:10.1002/smll.202508161

5. Duan B, Guo L, Yu Q, et al. Highly efficient solution-processed CZTSSe solar cells based on a convenient sodium-incorporated post-treatment method. *J Energy Chem*. 2020;40:196-203. doi:10.1016/j.jechem.2019.03.029

6. Mitzi DB, Gunawan O, Todorov TK, Wang K, Guha S. The path towards a high-performance solution-processed kesterite solar cell. *Sol Energy Mater Sol Cells*.

2011;95(6):1421-1436. doi:10.1016/j.solmat.2010.11.028

7. Zou L, Ma H, Zhu Q, et al. Defect regulation enhances the efficiency of Cu2ZnSnS4 solar cells by solution engineering. *Sol Energy Mater Sol Cells*. 2025;285(February):113555. doi:10.1016/j.solmat.2025.113555

8. Suckow S, Pletzer TM, Kurz H. Fast and reliable calculation of the two-diode model without simpli fi cations. *Prog Photovoltaics Res Appl*. 2014;22(2014):494-501. doi:10.1002/pip

9. Krustok J, Timmo K, Kauk-Kuusik M, Grossberg-Kuusk M. Tunneling-enhanced interface recombination and current loss curves in kesterite solar cells. *Appl Phys Lett*. 2023;123:1-5. doi:10.1063/5.0175082

10. Krustok J, Josepson R, Raadik T, Danilson M. Potential fluctuations in Cu2ZnSnSe4 solar cells studied by temperature dependence of quantum efficiency curves. *Phys B Condens Matter*. 2010;405(15):3186-3189. doi:10.1016/j.physb.2010.04.041

11. Troviano M, Taretto K. Analysis of internal quantum efficiency in double-graded bandgap solar cells including sub-bandgap absorption. *Sol Energy Mater Sol Cells*. 2011;95(3):821-828. doi:10.1016/j.solmat.2010.10.028

12. Kask E, Krustok J, Giraldo S, Neuschitzer M, López-Marino S, Saucedo E. Temperature dependent electrical characterization of thin film Cu 2 ZnSnSe 4 solar cells. *J Phys D Appl Phys*. 2016;49(8):085101. doi:10.1088/0022-3727/49/8/085101

13. Jian Y, Zhao Y, Zhao J, et al. Controllable SnS-Vapor-Assisted Selenization to Enhance Carrier Transport for Highly Efficient Kesterite Solar Cells. *Adv Energy Mater*. 2025;15(43):1-12. doi:10.1002/aenm.202504071

14. Kask E, Raadik T, Grossberg M, Josepson R, Krustok J. Deep defects in Cu 2ZnSnS 4 monograin solar cells. *Energy Procedia*. 2011;10:261-265. doi:10.1016/j.egypro.2011.10.188

15. Dolcet Sadurni M, Timmo K, Mikli V, et al. Effects of cationic substitution on the properties of Sb1-xBixSeI (x = 0–1) compounds. *J Alloys Compd*. 2025;1037(May). doi:10.1016/j.jallcom.2025.182292

16. Wu T, Chen S, Su Z, et al. Heat treatment in an oxygen-rich environment to suppress deep-level traps in Cu2ZnSnS4 solar cell with 11.51% certified efficiency. *Nat Energy*. 2025;10(5):630-640. doi:10.1038/s41560-025-01756-2

17. Luo Y, Chen G, Chen S, et al. Carrier Transport Enhancement Mechanism in Highly Efficient Antimony Selenide Thin-Film Solar Cell. *Adv Funct Mater*. 2023;33(14):1-11. doi:10.1002/adfm.202213941

18. Krustok J, Collan H, Yakushev M, Hjelt K. The Role of Spatial Potential Fluctuations in the Shape of the PL Bands of Multinary Semiconductor Compounds. *Phys Scr*. 1999;T79(1):179. doi:10.1238/Physica.Topical.079a00179
